# Prevalence of Endemic Respiratory Viruses During the COVID-19 Pandemic in Urban and Rural Malawi

**DOI:** 10.1093/ofid/ofad643

**Published:** 2023-12-21

**Authors:** Elen Vink, Louis Banda, Abena S Amoah, Stephen Kasenda, Jonathan M Read, Chris Jewell, Brigitte Denis, Annie Chauma Mwale, Amelia Crampin, Catherine Anscombe, Mavis Menyere, Antonia Ho

**Affiliations:** MRC-University of Glasgow Centre for Virus Research, Glasgow, UK; Malawi Epidemiology and Intervention Research Unit, Lilongwe and Chilumba, Malawi; Malawi Epidemiology and Intervention Research Unit, Lilongwe and Chilumba, Malawi; Department of Population Health, Faculty of Epidemiology and Population Health, London School of Hygiene and Tropical Medicine, London, UK; Leiden University Medical Center, Leiden, the Netherlands; Malawi Epidemiology and Intervention Research Unit, Lilongwe and Chilumba, Malawi; Centre for Health Information Computation and Statistics, Lancaster Medical School, Lancaster University, Lancaster, UK; Department of Mathematics and Statistics, Lancaster University, Lancaster, UK; Malawi-Liverpool-Wellcome Trust Clinical Research Programme, Blantyre, Malawi; Public Health Institute of Malawi, Lilongwe, Malawi; Malawi Epidemiology and Intervention Research Unit, Lilongwe and Chilumba, Malawi; Department of Population Health, Faculty of Epidemiology and Population Health, London School of Hygiene and Tropical Medicine, London, UK; School of Health and Wellbeing, University of Glasgow, Glasgow, UK; Department of Mathematics and Statistics, Lancaster University, Lancaster, UK; Liverpool School of Tropical Medicine, University of Liverpool, Liverpool, UK; Malawi-Liverpool-Wellcome Trust Clinical Research Programme, Blantyre, Malawi; MRC-University of Glasgow Centre for Virus Research, Glasgow, UK

**Keywords:** COVID-19, epidemiology, Malawi, respiratory viruses, community surveillance

## Abstract

**Background:**

We investigated endemic respiratory virus circulation patterns in Malawi, where no lockdown was imposed, during the COVID-19 pandemic.

**Methods:**

Within a prospective household cohort in urban and rural Malawi, adult participants provided upper respiratory tract (URT) samples at 4 time points between February 2021 and April 2022. Polymerase chain reaction (PCR) was performed for SARS-CoV-2, influenza, and other endemic respiratory viruses.

**Results:**

1626 URT samples from 945 participants in 542 households were included. Overall, 7.6% (n = 123) samples were PCR- positive for >1 respiratory virus; SARS-CoV-2 (4.4%) and rhinovirus (2.0%) were most common. No influenza A virus was detected. Influenza B and respiratory syncytial virus (RSV) were rare. Higher virus positivity were detected in the rural setting and at earlier time points. Coinfections were infrequent.

**Conclusions:**

Endemic respiratory viruses circulated in the community in Malawi during the pandemic, though influenza and RSV were rarely detected. Distinct differences in virus positivity and demographics were observed between urban and rural cohorts.

## Teaser

- Continued circulation of endemic respiratory viruses in Malawi during the pandemic.
- Influenza/RSV rarely detected.
- Higher virus positivity detected in the rural setting and at earlier time points.
- Coinfections infrequent.
- Distinct differences in demographics between urban and rural cohorts.

Acute respiratory infections are a leading cause of morbidity and mortality in Malawi [1], and the COVID-19 pandemic has drawn attention to viruses as an important cause of respiratory illness globally. However, due to competing health priorities, plus insufficient health care infrastructure and resources (including laboratory testing), Malawi's national surveillance system for acute respiratory infections is limited. Thus, the prevalence of SARS-CoV-2 and other respiratory viral infections in Malawi, like many other countries, is unknown. This is reflected by the high seroprevalence observed in recent SARS-CoV-2 serosurveys, implying substantially higher levels of transmission than what confirmed figures suggest [2–4].

**Figure 1.**
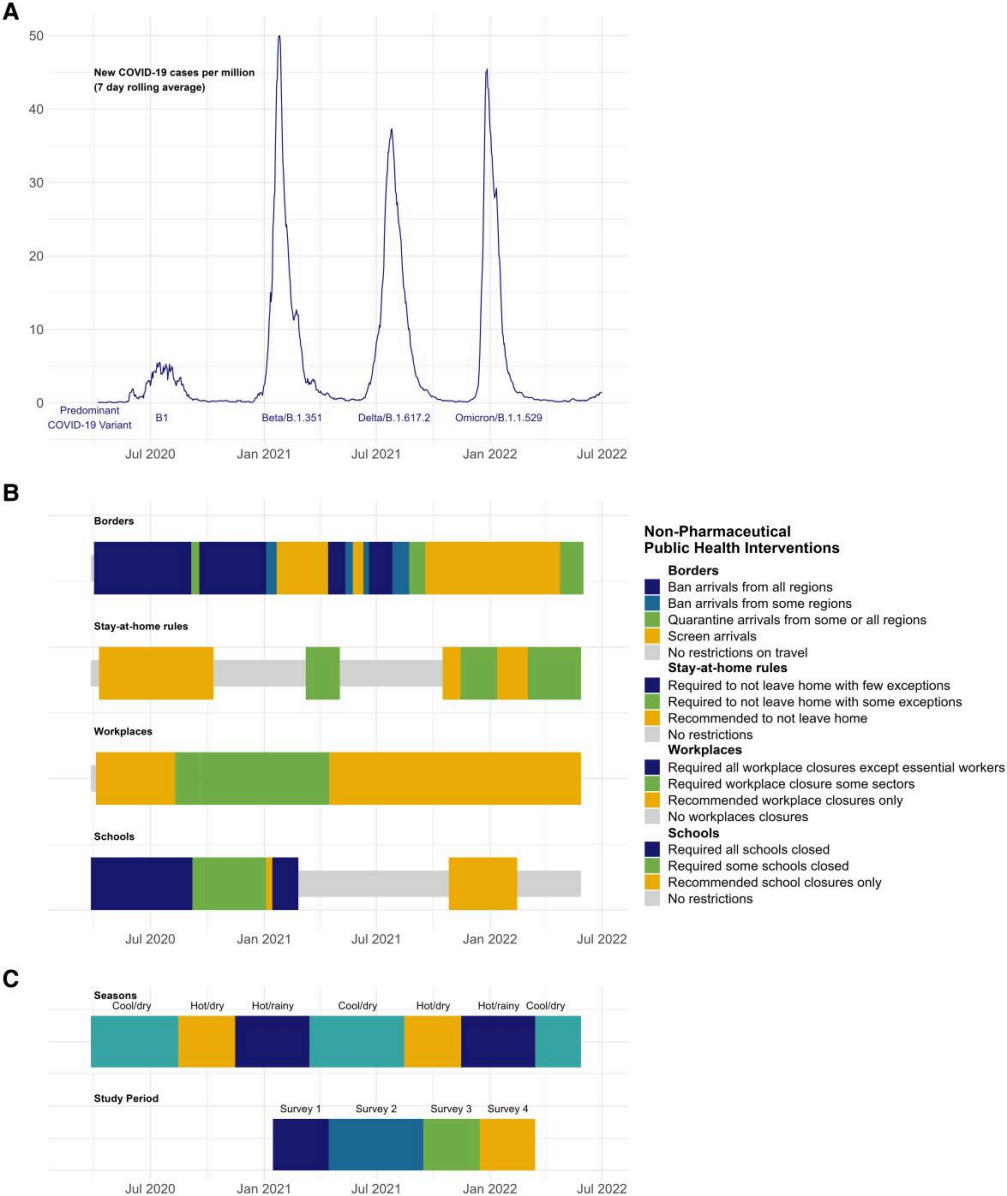
Timeline in Malawi: *A*, epidemic curve of SARS-CoV-2 infection. Data taken from Our World in Data [8]; *B*, nonpharmaceutical public health interventions. Data taken from Reuters COVID tracker [9]; *C*, seasons (data taken from the Department of Climate Change and Meteorological Services, Malawi) [10] and study periods.

Many countries, such as South Africa and the United Kingdom [5, 6], reported reduced and disrupted circulation of endemic respiratory viruses during the COVID-19 pandemic, with influenza viruses all but disappearing globally in 2020 to 2021 [7]. This is likely due to the impact of various nonpharmaceutical interventions (NPIs) on virus transmission, such as travel restrictions, stay-at-home orders, and school closures, though interactions between viruses may also play a role. In Malawi, SARS-CoV-2 was first detected on 2 April 2020 and has since caused 4 significant waves of infection (Figure 1*A*). A substantial number of NPIs were implemented in Malawi, including curfews and mask mandates, as well as the closures of schools, markets, hospitality, and borders (Figure 1*B*). However, NPIs in Malawi were less restrictive than many countries because a planned national lockdown was not implemented due to predicted negative socioeconomic consequences [8, 11]. The epidemiology of viral respiratory infections in Malawi may differ from countries with stricter NPIs.

Studies from other African countries, before and during the pandemic, have concentrated on urban areas, pediatric populations, or hospitalized cohorts. However, the majority population is adult and living in rural locations [12], and differences in the epidemiology and disease severity of several infectious diseases between urban and rural settings in Africa have been described [13, 14]. Additionally, the majority of those with respiratory viral infections do not present to medical services, preferring to self-medicate or consult a traditional healer [15, 16]. Some are asymptomatic.

Our study aims to address this gap through characterizing respiratory viruses from upper respiratory tract (URT) samples collected from a longitudinal community cohort of adults in urban and rural settings in Malawi during the COVID-19 pandemic. This knowledge is vital to guide national influenza and COVID-19 vaccine policy, inform clinical and diagnostic decision making in acute respiratory infection, and understand the impact that less stringent NPIs had on respiratory virus circulation patterns.

## METHODS

### Study Setting and Approvals

The COVSERO study was a prospective longitudinal serosurveillance cohort of randomly selected households in an urban location and a rural location in Malawi, conducted between February 2021 and April 2022. Malawi has a population of approximately 20 million with a median age of 18.1 years; 82% live in rural settings [17]. The climate is subtropical with 3 distinct seasons; hot/rainy (December–March), cool/dry (April–August), and hot/dry (September–November) [10] (Figure 1*C*). There is no routine influenza vaccination in Malawi. SARS-CoV-2 vaccination became available in March 2021.

Ethics approval was given by the research ethics committees of the Malawi College of Medicine (P11/20/3177) and the University of Glasgow College of Medicine, Veterinary, and Life Sciences (200200056).

### Study Participants

Participants were recruited between 24 February and 8 June 2021 (post–Beta wave) from randomly selected households in a geographically defined area of urban Lilongwe (Area 25; population 107 000) and the rural southern Karonga District (Karonga Health and Demographic Surveillance Site; population 51 000), as previously described [4]. Written informed consent was obtained for adults (age ≥18 years) and parental consent for those <18 years old.

Interviewers administered a baseline questionnaire capturing demographics, medical history, COVID-19 vaccination history, socioeconomic indicators, symptom histories, prevention behaviors, and recent exposures (Supplementary Table 1). An appropriate adult could choose to respond on behalf of those participants <18 years. HIV status was self-reported. An additional questionnaire collected household socioeconomic indicators (Supplementary Table 2).

Three-monthly follow-up was undertaken at 3 time points: survey 2, 28 June to 13 September 2021 (during Delta wave); survey 3, 4 October to 13 December 2021 (post–Delta wave); and survey 4, 27 January to 22 April 2022 (post–Omicron BA.1/2 wave; Figure 1*C*).

A subset of adult participants (50% of those interviewed in survey 1% and 100% in surveys 2–4) provided supervised self-obtained combined nasal and throat swabs, irrespective of symptom status, to assess for active infection of SARS-CoV-2 and other respiratory viruses.

### Sample Selection and Viral Real-time Polymerase Chain Reaction

Swabs were placed in viral transport medium (COVIDSafe Virus Inactivation Kit; HiMedia Labs) and stored at −80 °C prior to testing. Sample aliquots underwent SARS-CoV-2 real-time polymerase chain reaction (RT-PCR) testing at the Public Health Institute of Malawi laboratory; they were extracted via the m2000sp instrument and tested on the m2000rt system with a SARS-CoV-2 assay (all from Abbott). A cycle threshold ≤40 was considered positive.

We randomly selected 1636 samples at the individual level covering all 4 surveys at both sites to further test for adenovirus, bocavirus, enterovirus, human metapneumovirus, influenza viruses A to C, parainfluenza viruses 1 to 4, parechovirus, rhinovirus, and respiratory syncytial viruses (RSVs) A and B by multiplex RT-PCR (in-house assay designed at the Malawi-Liverpool-Wellcome Trust Clinical Research Programme; Supplementary Table 3). Samples were extracted via the Mag-Bind Universal Pathogen 96 Kit (Omega) with the Kingfisher Apex (Thermo Fisher). Positive and negative controls for all viral targets were included in all PCR runs. RNase P was used as an internal control. RT-PCR was performed on an Applied Biosystems ViiA7 RT-PCR System. A cycle threshold <40 was considered positive. Ten samples were excluded from analysis due to repeatedly failing internal controls or insufficient sample or metadata.

### Statistical Analysis

Categorical variables were reported as frequencies and percentages. Continuous variables were calculated as mean and SD or median and IQR based on normality of distribution. Individual and household demographic differences between the urban and rural cohorts were investigated by chi-square or Fisher exact test for categorical variables and Kruskal-Wallis test for continuous variables. *P* ≤ .05 was considered statistically significant. Binomial confidence intervals were calculated for proportions.

NPI score was calculated as the number of NPIs adopted in the 2 weeks preceding sampling (Supplementary Table 4). The crowding index was calculated as the number of people per sleeping rooms in a household. The asset score was calculated as the total estimated value (2014) of working items possessed by a household. The behavior score was calculated as the number of exposure risk activities undertaken in the week preceding sampling.

We used mixed-effects logistic regression to identify factors associated with PCR positivity (including and excluding SARS-CoV-2). We included the hierarchical random intercepts of participant nested within household to account for repeated testing of an individual and household clustering. We performed hypothesis-driven regression models, including the fixed effects of age, location, and time point within the study (chosen a priori). We also used a forward selection process to explore additional factors (Supplementary Table 5), which led to the inclusion of 2 fixed effects: (1) “attendance at place of work in the past week” in the final “including SARS-CoV-2” model and (2) “public-facing occupation” in the final “excluding SARS-CoV-2” model (Supplementary Table 6).

Differences in symptomatic proportions were tested by chi-square or Fisher exact test depending on expected frequencies. Odds of respiratory virus positivity based on symptoms were calculated by multivariable logistic regression analysis.

We analyzed temporal trends in viral species richness by location (number of viral species detected per population tested) via a generalized additive model with Poisson-distributed error structure. We aggregated the sample data by month of collection, included month of sampling as a spline term and an offset of the logged total monthly sample number. To assess spatial correlation, we constructed spatial variograms and compared them with a null model of the expected variance. For a given spatial scale, the expected variance was the 95% quantiles generated by randomly reassigning locations to samples.

All analyses were performed with R version 4.2.1.

## RESULTS

### Participant Characteristics

This analysis comprised 1626 URT samples from 945 participants in 542 households: Karonga, 714 samples from 463 participants in 251 households; Lilongwe, 912 samples from 482 participants in 291 households. These samples were collected between 24 February 2021 and 18 March 2022, with 335 samples from survey 1, 364 from survey 2, 493 from survey 3, and 434 from survey 4 (Table 1). An overall 442 participants contributed 1 sample, 348 contributed 2 samples, 132 contributed 3 samples, and 23 contributed 4 samples.

**Table 1.** Baseline Characteristics of Samples, Individuals, and Households by Site

	All	Karonga (Rural)	Lilongwe (Urban)	*P* Value^a^
Sample characteristics				
No.	1626	714 (43.9)	912 (56.1)	
Survey				
1 (24 Feb–8 Jun 2021)	335 (20.6)	163 (22.8)	172 (18.9)	
2 (28 Jun–13 Sept 2021)	364 (22.4)	92 (12.9)	272 (29.8)	
3 (4 Oct–13 Dec 2021)	493 (30.3)	247 (34.6)	246 (27.0)	
4 (27 Jan–22 Apr 2022)	434 (26.7)	212 (29.7)	222 (24.3)	
Season				
Hot/rainy 2020/21 (Dec–Mar)	168 (10.3)	83 (11.6)	85 (9.3)	
Cool/dry 2021 (Apr-Aug)	526 (32.3)	168 (23.5)	358 (39.3)	
Hot/dry 2021 (Sept-Nov)	442 (27.2)	202 (28.3)	240 (26.3)	
Hot/rainy 2021/22 (Dec-Mar)	490 (30.1)	261 (36.6)	229 (25.1)	
Individual characteristics				
No.	945	463 (49.0)	482 (51.0)	
Sex: female	556 (58.8)	232 (50.1)	324 (67.2)	<.01
Age, y				.26
15–39	554 (58.6)	273 (59.0)	281 (58.3)	
40–59	288 (30.5)	147 (31.7)	141 (29.3)	
≥60	103 (10.9)	43 (9.3)	60 (12.4)	
Median (IQR)	36.0 (25.5–48.2)	35.3 (24.1–48.2)	36.6 (27.0–48.3)	.09
Comorbidities				
Diabetes	16 (1.7)	0 (0.0)	16 (3.3)	<.01
Hypertension	107 (11.3)	37 (8.0)	70 (14.5)	<.01
Asthma	60 (6.3)	40 (8.6)	20 (4.1)	<.01
Lung disease	3 (0.3)	1 (0.2)	2 (0.4)	1.00
Heart disease	16 (1.7)	14 (3.0)	2 (0.4)	<.01
Chronic kidney disease	4 (0.4)	4 (0.9)	0 (0.0)	.06
Stroke	5 (0.5)	4 (0.9)	1 (0.2)	.21
Tuberculosis (past or present)	17 (1.8)	6 (1.3)	11 (2.3)	.37
HIV infection	71 (7.5)	23 (5.0)	48 (10.0)	<.01
Cancer	4 (0.4)	1 (0.2)	3 (0.6)	.63
No. of comorbidities				.84
0	769 (81.4)	379 (81.9)	390 (80.9)	
1	157 (16.6)	74 (16.0)	83 (17.2)	
≥2	19 (2.0)	10 (2.2)	9 (1.9)	
COVID-19 vaccination status				
Vaccinated at survey 1	79 (8.4)	18 (3.9)	61 (12.7)	<.01
Vaccinated at survey 4^b^	273 (36.1)	137 (31.8)	136 (41.8)	<.01
Occupation type				<.01
Unwaged	554 (58.6)	358 (77.3)	196 (40.7)	
Irregular wage/piecework	249 (26.3)	75 (16.2)	174 (36.1)	
Regular wage/salary	142 (15.0)	30 (6.5)	112 (23.2)	
Outdoor worker	576 (61.0)	385 (83.2)	191 (39.6)	<.01
Public facing occupation	535 (56.7)	309 (66.7)	226 (47.0)	<.01
Highest level of education				<.01
None or primary only	422 (44.7)	258 (55.7)	164 (34.0)	
Secondary or higher	523 (55.3)	205 (44.3)	318 (66.0)	
NPI score (0–9)^c^ : median (IQR)	4.0 (2.0–7.0)	3.0 (2.0–6.0)	5.0 (3.0–8.0)	<.01
Household characteristics				
No.	542	251 (46.3)	291 (53.7)	
Crowding index category^d^				<.01
<1.5	175 (32.5)	114 (45.8)	61 (21.1)	
1.5–2.4	238 (44.2)	95 (38.2)	143 (49.5)	
≥2.5	125 (23.2)	40 (16.1)	85 (29.4)	
Household income (quintile)				.53
1 (lowest)	116 (21.6)	54 (21.7)	62 (21.5)	
2	108 (20.1)	49 (19.7)	59 (20.4)	
3	112 (20.8)	45 (18.1)	67 (23.2)	
4	104 (19.3)	50 (20.1)	54 (18.7)	
5 (highest)	98 (18.2)	51 (20.5)	47 (16.3)	
Asset score,^e^ median (IQR)	24.0 (15.0–82.0)	18.0 (15.0–33.0)	43.0 (19.5–92.0)	<.01

Data are presented as No. (%) unless noted otherwise.

Abbreviation: NPI, nonpharmaceutical intervention.

^a^Chi-square or Fisher exact test depending on expected frequencies >5 or <5, respectively.

^b^Loss-to follow-up at survey 4: n = 756 (Karonga, n = 431; Lilongwe, n = 325).

^c^NPI score: number of precautions taken in the 2 weeks prior to sampling from a list of 9: face mask, avoiding shaking hands, avoiding touching own face, maintaining 1-m social distancing, increased hand washing, avoiding people with cough or fever, staying at home, avoiding crowds, avoiding hospitals.

^d^Crowding index: number of people per number of sleeping rooms in a household.

^e^Asset score: total estimated 2014 value of working household possessions from list—watch or clock, radio, charcoal iron, sewing machine, mobile phone, mosquito net, mattress, bed, bicycle, canoe, oxcart, motorcycle, car, electrical household items, tape/CD player, fan, electric iron, television, or refrigerator.

The median age of participants was 36.0 years (IQR, 25.5–48.2); 556 (58.8%) were female (Table 1). There was a higher proportion of female participants in Lilongwe than Karonga (67.2% vs 50.1%, *P* < .001). Most participants reported no comorbidities (81.4%). However, Lilongwe participants had a higher prevalence of diabetes (3.3% vs 0%, *P* < .001), hypertension (14.5% vs 8.0%, *P* = .002), and HIV infection (10.0% vs 5.0%, *P* = .004), while Karonga participants had a higher prevalence of asthma (8.6% vs 4.1%, *P* = .005) and heart disease (3.0% vs 0.4%, *P* = .002).

Distinct differences were seen in educational and occupational demographics among urban and rural participants (Table 1). Participants from Karonga were more likely to undertake unwaged work (77.3% vs 40.7%, *P* < .001), work outdoors (83.2% vs 39.6%, *P* < .001), and have a public-facing occupation (66.7% vs 47.0%, *P* < .001) than those in Lilongwe. They were also less likely than urban participants to have attended secondary/higher education (44.3% vs 66.0%, *P* < .001).

Living conditions differed between urban and rural households (Table 1). Households in Lilongwe were more crowded than in Karonga (median [IQR] crowding index, 2.0 [1.5–2.5] vs 1.5 [1.0–2.0]; *P* < .001) but had higher median asset scores (43 [1.5–92.0] vs 24 [15.0–82.0], *P* < .001).

### Respiratory Viral PCR Results

For any respiratory viruses overall, we detected a percentage positivity of 7.6% (123/1626 URT samples; 95% CI, 6.3%–9.0%), with 12.3% (116/945; 95% CI, 10.3%–14.5%) participant positivity over the study period. SARS-CoV-2 was detected most frequently (4.4%; 95% CI, 3.4%–5.5%; n = 71), followed by rhinovirus (2.0%; 95% CI, 1.4%–2.8%; n = 33), adenovirus (0.6%; 95% CI, 0.3%–1.0%; n = 9), and bocavirus (0.3%; 95% CI, 0.1%–0.6%; n = 4). Influenza B and C viruses, parainfluenza virus, RSV, enterovirus, human metapneumovirus, and parechovirus were detected in small numbers (Supplementary Table 7). No influenza A viruses were detected.

**Figure 2.**
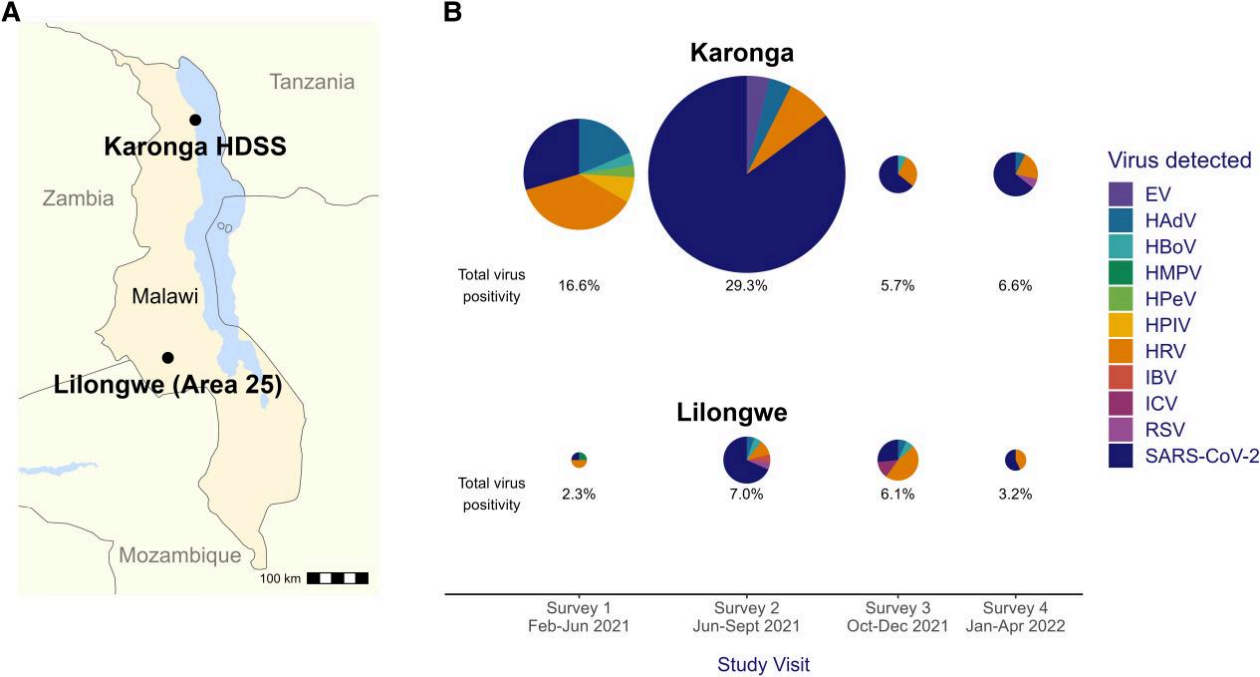
*A*, Map of study sites. *B*, Etiology of respiratory viral infection by location and study visit. Samples tested per survey/site: survey 1, Karonga (n = 163), Lilongwe (n = 172); survey 2, Karonga (n = 92), Lilongwe (n = 272); survey 3, Karonga (n = 247), Lilongwe (n = 246); survey 4, Karonga (n = 212), Lilongwe (n = 222). EV, enterovirus; HAdV, human adenovirus; HBoV, human bocavirus; HDSS, Health and Demographic Surveillance Site; HMPV, human metapneumovirus; HPeV, human parechovirus; HPIV, human parainfluenza viruses; HRV, human rhinovirus; IBV, influenza B virus; ICV, influenza C virus; RSV, respiratory syncytial virus.

In a mixed-effects analysis, respiratory virus percentage positivity was higher in rural Karonga (10.9%, 78/714) than urban Lilongwe (4.9%, 45/912; odds ratio [OR], 2.58; 95% CI, 1.37–4.91; Table 2, Supplementary Table 7, Figure 2). This association was also seen if SARS-CoV-2 positivity was excluded from the binary outcome (OR, 1.84; 95% CI, 1.04–3.24). No significant difference in virus positivity was found among age groups, including and excluding SARS-CoV-2.

**Table 2.** Risk Factors for Respiratory Virus Positivity: Mixed-Effects Logistic Regression Analyses

	PCR Positive for Any Respiratory Virus^a^			
	Including SARS-CoV-2		Excluding SARS-CoV-2	
	OR (95% CI)	*P* Value	OR (95% CI)	*P* Value
Site				
Lilongwe	1 [Reference]		1 [Reference]	
Karonga	**2.58 (1.35–4.91)**	**<**.**01**	**1.84 (1.04–3.24)**	.**04**
Season, year				
Hot/rainy 2020–2021 (Dec-Mar)	1 [Reference]		1 [Reference]	
Cool/dry 2021 (Apr-Aug)	0.85 (.39–1.85)	.69	0.51 (.24–1.09)	.08
Hot/dry 2021 (Sept-Nov)	0.48 (.21–1.08)	.08	0.46 (.21–1.02)	.06
Hot/rainy 2021–2022 (Dec-Mar)	**0.29 (.12–.69)**	**<.01**	**0.26 (.11–.62)**	**<**.**01**
Age, y				
15–39	1 [Reference]		1 [Reference]	
40–59	0.74 (.38–1.45)	.38	0.74 (.40–1.38)	.34
≥60	1.61 (.67–3.86)	.28	0.95 (.39–2.32)	.92
Attendance at place of work in the past week			…	…
No	1 [Reference]			
Yes	1.56 (.86–2.83)	.14		
Public-facing occupation	…	…		
No			1 [Reference]	
Yes			1.19 (.67–2.11)	.54

Univariable analysis: Supplementary Tables 10 and 11. Forward model selection process: Supplementary Table 5. Mixed-effects model summaries: Supplementary Table 6.

Abbreviations: OR, odds ratio; PCR, polymerase chain reaction.

^a^Bold indicates *P* ≤ .05.

**Figure 3.**
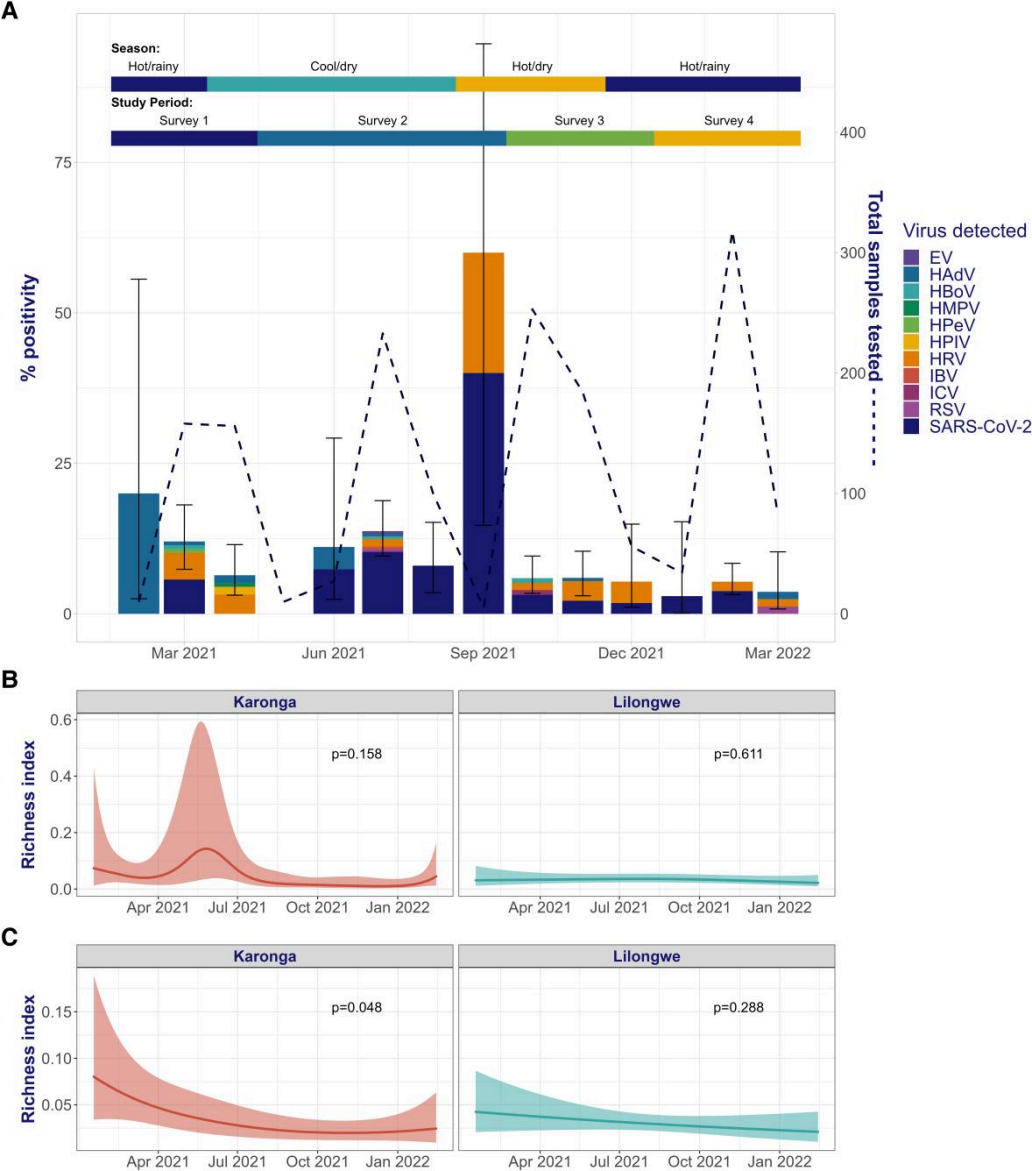
*A*, Etiology of respiratory viral infection by month. Error bars indicated 95% CI. Temporal trend in respiratory virus species richness (including SARS-CoV-2) by location, as predicted by the generalized additive model: *B*, including SARS-CoV-2; *C*, excluding SARS-CoV-2. EV, enterovirus; HAdV, human adenovirus; HBoV, human bocavirus; HMPV, human metapneumovirus; HPeV, human parechovirus; HPIV, human parainfluenza viruses; HRV, human rhinovirus; IBV, influenza B virus; ICV, influenza C virus; RSV, respiratory syncytial virus.

Respiratory virus positivity peaked at 11.9% (20/168) during the first season of the study (hot/rainy, 2020–2021), with a decline in positivity over subsequent seasons: cool/dry, 2021 (9.5%; mixed-effects OR, 0.85; 95% CI, .39–1.85), hot/dry, 2021 (6.6%; OR, 0.48; 95% CI, .21–1.08), hot/rainy, 2021–2022 (4.9%; OR, 0.29; 95% CI, .12–.69; Table 2, Supplementary Table 7). A similar pattern was seen when SARS-CoV-2 positivity was excluded from the analysis. SARS-CoV-2 positivity peaked at 6.1% during the cool/dry season of 2021 coinciding with the Delta variant–driven wave of infection in Malawi. There was no consistent change by season in the proportion of participants who were symptomatic or who attended a health care facility. There was a trend toward reduced species richness in respiratory viruses (excluding SARS-CoV-2) over the study period, particularly in Karonga (Figure 3).

Two or more respiratory viruses were detected in 3 (0.2%) samples: 2 dual infections (SARS-CoV-2 with rhinovirus and SARS-CoV-2 with enterovirus) and 1 triple infection (SARS-CoV-2, rhinovirus, and adenovirus). All coinfections were detected in Karonga participants during the first 2 surveys.

Concurrent infections with the same respiratory virus were detected within 8 households. SARS-CoV-2 was identified in 2 household members of 6 households and in 3 members of 1 household. Adenovirus was detected in 2 members of 1 household. Infection with the same respiratory virus in cohabiting participants at consecutive study visits was detected in 4 households: SARS-CoV-2 (n = 3) and rhinovirus (n = 1). Of note, SARS-CoV-2 was detected in 1 participant at the 3 consecutive time points: March, July, and October 2021 in a >60-year-old male with diabetes who received 2 doses of Oxford AstraZeneca COVID-19 vaccine between the first and second study visits. This participant reported COVID-19–related symptoms at all 3 time points but with decreasing symptoms over time (Supplementary Table 8).

Correlating respiratory viral PCR results with symptoms, we found that 39.4% (641/1626) of samples were collected from participants who reported ≥1 respiratory virus-related symptoms in the 2 weeks preceding sampling. A higher proportion of participants who were virus positive reported symptoms (50.4% [62/123] vs 38.7% [579/1498], *P* = .01) and sought medical attention (15.4% [19/123] vs 8.7% [131/1503], *P* = .02) when compared with participants who were virus negative. In multivariable analysis, rhinorrhea (OR, 3.41; 95% CI, 1.90–6.19) and wheeze (OR, 3.77; 95% CI, 1.24–10.69) were associated with increased odds of testing PCR positivity (Supplementary Table 9). There was no significant difference in the proportion of participants with symptoms between those infected with SARS-CoV-2 and those with other endemic respiratory viruses (52.9% vs 44.2%, *P* = .45). However, a significantly higher proportion of participants who were SARS-CoV-2 positive reported fever (17.6% vs 3.8%, *P* = .02), myalgia (19.1% vs 0%, *P* = .001), and joint pain (16.2% vs 3.8%, *P* = .04) as compared with those who were positive for other respiratory viruses. Only 1.4% (22/1626) of participants reported symptoms fulfilling the World Health Organization criteria for an “influenza-like illness” (ILI) [18]; of these, 13.6% were virus positive, all with SARS-CoV-2. A higher proportion of participants who were respiratory virus positive sought medical attention (15.4% vs 8.7%, *P* = .02). Two participants were admitted to hospital; both were PCR negative.

We found no evidence of spatial correlation among participants positive for any respiratory virus, a respiratory virus excluding SARS-CoV-2, rhinovirus, or adenovirus (Supplementary Figure 1). Other respiratory viruses were detected in insufficient numbers to be analyzed individually.

## DISCUSSION

Our longitudinal cohort study demonstrated that endemic respiratory viruses continued to circulate in the community in Malawi during the COVID-19 pandemic. Higher virus positivity was detected in the rural setting vs the urban setting, and the prevalence of respiratory viral infections declined over the study period. Influenza A was absent; influenza B and RSV were rarely detected; and coinfections were infrequent. These results are consistent with studies from across the world [6, 7, 19, 20] that reported low influenza and RSV positivity in the 2 years following the emergence of SARS-CoV-2, likely due to NPI implementation.

Few studies in Africa have evaluated the circulation of other respiratory viruses during the COVID-19 pandemic. ILI and severe acute respiratory illness (SARI) surveillance studies in Zambia and Madagascar detected no influenza viruses during 2020 [21–23], while South African national surveillance detected only 1 case of influenza A virus between mid-April 2020 and September 2021 [24, 25]. In contrast, ILI and SARI surveillance studies from Ghana [26], The Gambia [27], and the Democratic Republic of Congo [28] demonstrated ongoing transmission of influenza A and B during 2020 and 2021, with all 3 studies detecting the highest levels of influenza positivity in children. Comparison with prepandemic data from Ghana [26] demonstrated a decline in influenza positivity during 2020 to 2021, whereas data from Zambia showed an absence of influenza in 2020 but a resurgence to prepandemic levels in 2021 [23].

Most African studies that analyzed RSV activity during the pandemic noted a complete absence of RSV activity [28], low positivity [21, 23, 27, 29], or significantly disrupted circulation patterns [30]. Conversely, a Madagascan study revealed no change to the epidemiologic profile of RSV between 2018 and 2022 [22].

In contrast to the studies cited, we sampled individuals in the community irrespective of symptomatology, rather than those presenting to health facilities with acute respiratory illness, thus circumventing any bias introduced by health care–seeking behavior. This likely explains our lower levels of influenza and RSV positivity, since detection of both viruses has been shown to be strongly associated with symptomatic illness (pathogen-attributable fractions >80%) [31, 32], suggesting that they are infrequently detected in asymptomatic individuals. Additionally, since URT samples were self-collected, we opted not to sample children <15 years of age, who tend to have higher positivity for influenza [21, 22, 26–28, 30] and RSV [21, 22, 30] than adults.

After SARS-CoV-2, rhinovirus was the most frequently detected virus in our study and was present throughout the study period. This has been reported in many countries, such as the United States [33], United Kingdom [34], and Australia [35], and has been associated with the reopening of schools [35–37]. Studies in Africa that tracked rhinovirus prevalence [21–23, 27] found similar rates of rhinovirus positivity before and during the pandemic. Persistent circulation of adenovirus during the pandemic was noted by studies from the United States, United Kingdom, and Zambia [21, 23, 33, 34] and was also observed in our study.

The varying impact of the pandemic on the circulation of different viruses may be influenced by a variety of virologic, environmental, and behavioral factors. In our study, the participants who were PCR positive and most likely to be asymptomatic were those positive for adenovirus or rhinovirus, in keeping with studies demonstrating a lower attributable fraction for these viruses [31]. Asymptomatic individuals may have adhered less to NPIs, with a consequent higher risk of onward transmission. Intrinsic structural differences may play a key role: adenovirus and rhinovirus are nonenveloped viruses, while influenza and RSV are enveloped. Enveloped viruses survive for shorter periods on surfaces [38] and are less resistant to alcohol hand gels (although not soap) [39, 40], making their transmission more susceptible to disruption by NPIs.

The absence/low level of influenza A/B and RSV in Malawi despite limited community-based NPIs suggests that international air travel may be a key factor affecting seasonal virus transmission. Interestingly, the reemergence of influenza viruses in Madagascar in July 2021 closely followed the resumption of international flights, though linkage to imported cases is not yet confirmed [22]. Genomic studies have suggested that East/Southeast Asia [41] and West Africa [42] are key reservoirs of sustained, rather than seasonal, influenza circulation, from which antigenic variants emerge. However, with restrictions on international/cross-border travel, transmission routes are limited or cut, thus curtailing global spread. Rhinovirus, though, maintains local community reservoirs, particularly in children [43], resulting in persistent circulation that is not reliant on international spread.

Viral interference at a cellular level is likely to influence the circulation of viruses at a population level. Rhinovirus infection has been shown to inhibit coinfection/subsequent infection with SARS-CoV-2 [44] and influenza A [45]. Further studies are required to tease out the interplay among all these different factors.

A higher prevalence of SARS-CoV-2 and endemic viruses was detected in rural Karonga than urban Lilongwe during the study (Figure 2). This is likely due to a combination of factors. In 2020, a sizable prevalence of SARS-CoV-2 was detected in urban areas of Zambia, but none was detected by a surveillance study or hospital testing in rural southern Zambia [21], suggesting that the first wave of SARS-CoV-2 failed to infiltrate this rural area. Our study covered a later period and may therefore have captured the point at which SARS-CoV-2 penetrated and spread in rural communities in Malawi (Delta variant–driven third wave). The rural cohort adhered less to NPIs (Table 1), which likely facilitated community virus circulation. Furthermore, the circulation of respiratory viruses in Karonga could have been bolstered by cross-border virus transmission due to proximity to the Tanzanian border, where NPIs were less stringently enforced than in Malawi [8].

Metadata from our urban and rural cohorts illustrated substantial demographic differences between these communities; thus, subnational variations in respiratory virus epidemiology are unsurprising [46–49]. These differences highlight the importance of including urban and rural sites in surveillance studies, which is a strength of our study. Other strengths are the comprehensive metadata associated with each sample, a study period covering multiple waves of SARS-CoV-2 infection, testing for a broad range of respiratory viruses, and the ability to capture concurrent infections within households plus recurrent or potentially chronic infection in an individual.

Our study had some limitations. First, we did not have prepandemic community data with which to compare our findings. Second, we did not collect respiratory samples from children, in whom incidence of respiratory virus infections, particularly RSV, is known to be higher. The absence of respiratory viral infection status in children in the household may have affected our assessment of household-level variables associated with adult PCR positivity. Of note, our data did demonstrate that a higher proportion of participants with a symptomatic child aged <5 years in their household tested positive for respiratory viruses (11.8% vs 7.2%; Supplementary Tables 10 and 11). Finally, we recruited from 2 sites in Malawi; consequently, our results may not be representative of the whole country.

## CONCLUSION

Our study demonstrates that respiratory viruses continued to circulate in Malawi during the COVID-19 pandemic. However, the prevalence of certain viruses was low or absent, particularly enveloped viruses such as influenza A/B and RSV. Distinct differences in demographic factors and virus positivity were observed between urban and rural cohorts, highlighting the importance of including diverse communities in studies of respiratory virus epidemiology. Further studies are needed to assess the ongoing impact of cocirculation of SARS-CoV-2 with endemic respiratory viruses at patient and population levels in Malawi.

## Supplementary Data

Supplementary materials are available at *Open Forum Infectious Diseases* online. Consisting of data provided by the authors to benefit the reader, the posted materials are not copyedited and are the sole responsibility of the authors, so questions or comments should be addressed to the corresponding author.

## Supplementary Material

ofad643_Supplementary_Data

## Notes

***Acknowledgments.*** Thank you to the participants for taking part in this study and providing these samples. Also, we are very grateful to the COVSERO research and laboratory team at the Malawi Epidemiology and Intervention Research Unit and Malawi-Liverpool-Wellcome Trust Clinical Research Programme for their hard work in collecting the study data and processing the samples.

***Author contributions.*** Conceptualization: E. V., A. H., A. C. M. Data curation: E. V., A. H. Formal analysis: E. V. Funding acquisition: A. H., A. C. Investigation: L. B., A. S. A., S. K., C. J., B. D., C. A., M. M. Methodology: E. V., C. A., M. M., A. H. Project administration: S. K., B. D. Software: E. V., J. M. R. Supervision: A. H., J. M. R., A. C. Visualization: E. V. Writing–original draft: E. V., A. H. Writing–review and editing: all authors.

***Data presentation.*** Preliminary results from this study were presented as posters at the Kamazu University of Health Sciences Research Dissemination Conference, Blantyre, Malawi, 24 November 2022, and the Association of Physicians of Great Britain and Ireland Annual Meeting, Liverpool, United Kingdom, 21 April 2023. Final results were presented as posters at the UK Pandemic Sciences Network Meeting, Liverpool, United Kingdom, 5 September 2023, and the Seventh African Network for Influenza Surveillance and Epidemiology Meeting, Johannesburg, South Africa, 11 September 2023.

***Data sharing.*** Individual pseudo-anonymized data and accompanying documentation can be made available on reasonable request: requesters will need to fill and sign a data transfer agreement and should contact info@meiru.mw quoting the title of the article in the first instance.

***Patient consent statement.*** Ethics approval was given by the research ethics committees of the Malawi College of Medicine (P11/20/3177) and the University of Glasgow College of Medicine, Veterinary and Life Sciences (200200056). Patients' written informed consent was obtained prior to the collection of metadata and biological samples.

***Financial support*.** This work was supported by grants from the Wellcome Trust (217073/Z/19/Z and 221989/Z/20/Z); the Medical Research Foundation (MRF-160-0018-ELP-HO-C0910); and the Medical Research Council (MC_ST_CVR_2019 and MC_UU_00034/6).
