# Correction to: Prevalence of Endemic Respiratory Viruses During the COVID-19 Pandemic in Urban and Rural Malawi

**DOI:** 10.1093/ofid/ofag682

**Published:** 2026-10-13

This is a correction to: Elen Vink, Louis Banda, Abena S Amoah, Stephen Kasenda, Jonathan M Read, Chris Jewell, Brigitte Denis, Annie Chauma Mwale, Amelia Crampin, Catherine Anscombe, Mavis Menyere, Antonia Ho, Prevalence of Endemic Respiratory Viruses During the COVID-19 Pandemic in Urban and Rural Malawi, *Open Forum Infectious Diseases*, Volume 11, Issue 2, February 2024, ofad643, https://doi.org/10.1093/ofid/ofad643

Post-publication, the authors wish to add the following acknowledgement to this article:

“We wish to acknowledge Shannon McSweeney, whose assay development work shaped the methodology that was used in the PCR testing in this manuscript.”

These details have been added only in this correction notice to preserve the published version of record.

