# Supplementary material for: Prevalence of Endemic Respiratory Viruses During the COVID-19 Pandemic in Urban and Rural Malawi
Source: Open Forum Infect Dis. 2023 Dec 21;11(2):ofad643. doi: 10.1093/ofid/ofad643 (PMC10836885; doi:10.1093/ofid/ofad643)

Supplementary material

Table S1: COVSERO Individual Questionnaire

| **SECTION 1. IDENTIFICATION** | | | | | | | | | |
| --- | --- | --- | --- | --- | --- | --- | --- | --- | --- |
| 1.1 | Study site | | | | | 1= Karonga | | | |
|  |  | | | | | 2=Lilongwe | | | |
| 1.2 | Study ID | | | | | Study ID | | | |
|  |  |  |  |  |  | barcode | | | |
| 1.3 | CRSNO  *For Karonga only* | | | | | Reporting group  Cluster  House Number  Member Number | | | |
| 1.4 | Household number  *For Lilongwe* | | | | |  | | | |
| 1.5 | Contact number | | | | | Contact number barcode | | | |
| 1.6 | Sex | | | | | 0 = Male | | | |
|  |  |  |  |  |  | 1 = Female | | | |
| 1.7 | Date of birth | | | | | (dd/mm/yyyy) | | | |
|  |  |  |  |  |  |  |  |  |  |
| 1.8 | How old are you (age in years) | | | | | Age in years | | | |
|  |  |  |  |  |  |  |  |  |  |
| **SECTION 2: SOCIO-ECONOMIC INDICATORS**  ***(this section should be excluded in subsequent visits)*** | | | | | | | | | |
| 2.1 | Main Occupation | | | | | ______________________ | | | |
| 2.2 | Employment type | | | | | 0= unwaged  1= irregular wage/piecework  2= regular wage/salary | | | |
| 2.3 | Normal workplace setting / nature of work  ***Record all responses that apply.*** | | | | | 1= Outdoors  2=Factory/warehouse  3=Office  4=School (teacher)  5=Housework  6= (Mini)bus driver/conductor  7= Healthcare worker  10= Other (specify)  __________________________ | | | |
| 2.4 | Have you ever been to school? | | | | | 0= No  1= Currently  2= Ever | | | |
| 2.5 | If yes to ‘ever been to school or currently enrolled’, what is the highest level of education completed?  ***(Only applicable if the answer to 2.4 was “No”)*** | | | | | 1 = Primary  2 =Secondary,  3=Tertiary | | | |
| 2.6 | What was the highest form/standard completed? | | | | | form/standard | | | |
| **SECTION 3: MEDICAL HISTORY**  ***(this section should be excluded in subsequent visits Except Q3.1)*** | | | | | | | | | |
| 3.1 | Is there any chance that you might be pregnant?  (*if the answer to Q1.6 was “female”)* | | | | | 0= no  1= Yes  3=refused to answer  8= I do not know/ | | | |
| 3.2 | Have you ever been diagnosed with any of the following? *(choose as many as apply)* | | | | | | | | |
|  | A | | Diabetes mellitus | | 0=No  1=Yes, not on medication  2=yes, on medication | | | |  |
|  | B | | High blood pressure | | 0=No  1=Yes, not on medication  2=yes, on medication | | | |  |
|  | C | | Asthma | | 0=No  1=Yes, not on medication  2=yes, on medication | | | |  |
|  | D | | Chronic lung disease (not asthma) | | 0=No  1=Yes, not on medication  2=yes, on medication | | | |  |
|  | E | | Heart disease | | 0=No  1=Yes, not on medication  2=yes, on medication | | | |  |
|  | F | | Chronic kidney disease | | 0=No  1=Yes, not on medication  2=yes, on medication | | | |  |
|  | G | | Stroke | | 0=No  1=Yes, not on medication  2=yes, on medication | | | |  |
|  | H | | Tuberculosis (Past or present) | | 0=No  1=Yes, Previous treated for TB with 6-8 months of treatment  2=Currently treated for TB with planned 6-8months of treatments | | | |  |
|  | I | | Chronic Liver disease e.g. hepatitis, cirrhosis etc | | 0=No  1=Yes, not on medication  2=yes, on medication | | | |  |
|  | J | | HIV | | 0=No  1=Yes, not on medication  2=yes, currently taking ART for treatment of HIV | | | |  |
|  | K | | Cancer | | 0=No  1=Yes, not on medication  2=yes, on medication | | | |  |
|  | K1 | | Please specify the type of cancer | | Type of cancer | | | |  |
|  | L | | Other | | 0=No  1=Yes, not on medication  2=yes, on medication | | | |  |
| 3.2L2 | Please specify the disease  (if the answer to Q3.2L was “yes”) | | | | | Name of disease  ___________________________ | | | |
| 3.3 | What regular medication(s) are you on? | | | | | Name of regular medication _____________ | | | |
|  |  | | | | |  | | | |
| **SECTION 4: EXPOSURE HISTORY** | | | | | | | | | |
| 4.1 | How many hours did you spend inside your house from this time yesterday up until now (including sleeping)  *This does not include time spent* ***at home*** ***but outside*** | | | | | 1= < 5 hrs | | | |
|  |  |  |  |  |  | 2= 5-8 Hrs | | | |
|  |  |  |  |  |  | 3= 9-13 hrs | | | |
|  |  |  |  |  |  | 4= 14-19 hrs | | | |
|  |  |  |  |  |  | 5= ≥20 hrs | | | |
|  |  |  |  |  |  | 8= Don't know | | | |
| 4.2 | Have you been going to your regular place of work (office, factory, market to sell etc) since April this year? | | | | | 0=No  1= Yes, between April & July only  2= Yes, only after July  3=Yes, April to date | | | |
| 4.3 | Do you work mostly indoors or outdoors? | | | | | 0= outdoors  1=Indoors | | | |
| 4.4 | Does your work involve meeting or coming into contact with members of the public? (e.g., like a teacher, vendor, shop worker /owner or a nurse) | | | | | 0= no  1= Yes  8= I do not know/not sure | | | |
| 4.5 | How many times have you accessed the following facilities in the past 1 week ***(This question is valid for the first home visit only so skip to Question )*** | | | | | | | | |
|  | A1 | | Your office building/workplace/school | | | | ________(0-100) | |  |
|  | A2 | | ***(if the number was >0)***  Approximately how many people were you in the same room with when you were at your office building /workplace / school the last time you went? | | | | 1= 1–4  2= 5-9  3= 10-19  4= 20-29  5= 30 or more | |  |
|  | B1 | | Church / Religious Service / Prayer meeting | | | | ________(0-100) | |  |
|  | B2 | | Approximately how many people were present at this gathering the last time you went?  ***(if the number was >0)*** | | | | 1= 1–4  2= 5-9  3= 10-19  4= 20-29  5= 30 or more | |  |
|  | C1 | | Public transportation | | | | _________(0-50) | |  |
|  | C2 | | Approximately how many people did you typically meet the last time you used public transportation? ***(if the number was >0)*** | | | | 1= 1–4  2= 5-9  3= 10-19  4= 20-29  5= 30 or more | |  |
|  | D1 | | Clinic/hospital | | | | _______(0-100) | |  |
|  | D2 | | Approximately how many people did you typically meet during the last visit to the clinic / hospital?  ***(if the number was >0)*** | | | | 1= 1–4  2= 5-9  3= 10-19  4= 20-29  5= 30 or more | |  |
|  | E1 | | Market/shops | | | | ____ (0-100) | |  |
|  | E2 | | Approximately how many people were present at the market / shops when you visited last?  ***(if the number was >0)*** | | | | 1= 1–4  2= 5-9  3= 10-19  4= 20-29  5= 30 or more | |  |
|  | F1 | | Bar/drinking joint/night-club | | | | ____ (0-100) | |  |
|  | F2 | | Approximately how many people were present the last time you visited?  ***(if the number was >0)*** | | | | 1= 1–4  2= 5-9  3= 10-19  4= 20-29  5= 30 or more | |  |
|  | G1 | | Hairdresser /beauty salon | | | | _________(0-50) | |  |
|  | G2 | | Approximately how many people were present at the hairdresser / beauty salon when you visited last?  ***(if the number was >0)*** | | | | 1= 1–4  2= 5-9  3= 10-19  4= 20-29  5= 30 or more | |  |
|  | H1 | | Weddings / Engagements / Bridal showers | | | | ________(0-100) | |  |
|  | H2 | | Approximately how many people were present at this gathering the last time you went?  ***(if the number was >0)*** | | | | 1= 1–4  2= 5-9  3= 10-19  4= 20-29  5= 30 or more | |  |
|  | I1 | | Funerals | | | | ________(0-100) | |  |
|  | I2 | | Approximately how many people were present at this gathering the last time you went?  ***(if the number was >0)*** | | | | 1= 1–4  2= 5-9  3= 10-19  4= 20-29  5= 30 or more | |  |
|  | J1 | | Demonstrations | | | | ________(0-100) | |  |
|  | J2 | | Approximately how many people were present at this gathering the last time you went?  ***(if the number was >0)*** | | | | 1= 1–4  2= 5-9  3= 10-19  4= 20-29  5= 30 or more | |  |
|  |  | |  | | | |  | |  |
| 4.6 | Travel | | | | | | |  | |
|  | A | | In the past 1 week how many times did you travel outside out of your residential area but within this district? | | | | ______________ | |  |
|  | B | | In the past 1 week how many times did you travel outside this district? | | | | ______________ | |  |
|  |  | |  | | | |  | |  |
| **SECTION 5: PREVENTION BEHAVIOURS** | | | | | | | | | |
|  | In the past 2 weeks, which measures (if any) have you personally taken to reduce the spread of the coronavirus (i.e. COVID-19)? | | | | | | | | |
|  | A | | Wearing a face mask | | 0=No 1=Yes 8 Do not know | | | |  |
|  | B | | Avoided shaking hands | | 0=No 1=Yes 8 Do not know | | | |  |
|  | C | | Avoided touching my face | | 0=No 1=Yes 8 Do not know | | | |  |
|  | D | | Maintained safe distance of at least 1 metre | | 0=No 1=Yes 8 Do not know | | | |  |
|  | E | | Wash hands more often/ using hand sanitizer | | 0=No 1=Yes 8 Do not know | | | |  |
|  | F | | Covered nose and mouth when sneezing/coughing | | 0=No 1=Yes 8 Do not know | | | |  |
|  | G | | Avoided contact with people who have fever or cough | | 0=No 1=Yes 8 Do not know | | | |  |
|  | H | | Avoided going out in general | | 0=No 1=Yes 8 Do not know | | | |  |
|  | I | | Avoided crowded areas | | 0=No 1=Yes 8 Do not know | | | |  |
|  | J | | Avoided going to hospital | | 0=No 1=Yes 8 Do not know | | | |  |
|  | L | | Other (specify) | | _________________ | | | |  |
| **SECTION 6: SYMPTOM HISTORY** | | | | | | | | | |
| 6.1 | Have you experienced any of the following within the **past 2 weeks** (only relevant for the first visit) | | | | | | | | |
|  | A1 | | Severe or unusual Headache | | 0=no 1=yes  8= I do not know/remember | | | |  |
|  | | A2 | | If yes, the last time you had this symptom, how many days did it last? | | ________ | | | |
|  | B1 | | Feeling more tired than usual | | 0=no 1=yes  8= I do not know/remember | | | |  |
|  | | B2 | | If yes, the last time you had this symptom, how many days did it last? | | ________ | | | |
|  | C1 | | Fever (feeling extremely hot) | | 0=no 1=yes  8= I do not know/remember | | | |  |
|  | | C2 | | If yes, the last time you had this symptom, how many days did it last? | | ________ | | | |
|  | D1 | | Chills (feeling extremely cold / shivering) | | 0=no 1=yes  8= I do not know/remember | | | |  |
|  | | D2 | | If yes, the last time you had this symptom, how many days did it last? | | ________ | | | |
|  | E1 | | Runny/blocked nose | | 0=no 1=yes  8= I do not know/remember | | | |  |
|  | | E2 | | If yes, the last time you had this symptom, how many days did it last? | | ________ | | | |
|  | F1 | | Loss of smell | | 0=no 1=yes  8= I do not know/remember | | | |  |
|  | | F2 | | If yes, the last time you had this symptom, how many days did it last? | | ________ | | | |
|  | G1 | | Shortness of breath or difficulty breathing | | 0=no 1=yes  8= I do not know/remember | | | |  |
|  | | G2 | | If yes, the last time you had this symptom, how many days did it last? | | ________ | | | |
|  | H1 | | wheezing | | 0=no 1=yes  8= I do not know/remember | | | |  |
|  | | H2 | | If yes, the last time you had this symptom, how many days did it last? | | ________ | | | |
|  | I1 | | New, persistent cough | | 0=no  1= yes non-productive  2=yes productive  3=yes with blood  8=unknown | | | |  |
|  | | I2 | | If yes, the last time you had this symptom, how many days did it last? | | ________ | | | |
|  | J1 | | Nausea and/or vomiting | | 0=no 1=yes  8= I do not know/remember | | | |  |
|  | | J2 | | If yes, the last time you had this symptom, how many days did it last? | | ________ | | | |
|  | K1 | | Loss of appetite | | 0=no 1=yes  8= I do not know/remember | | | |  |
|  | | K2 | | If yes, the last time you had this symptom, how many days did it last? | | ________ | | | |
|  | L1 | | Loss of taste | | 0=no 1=yes  8= I do not know/remember | | | |  |
|  | | L2 | | If yes, the last time you had this symptom, how many days did it last? | | ________ | | | |
|  | M1 | | Sore throat | | 0=no 1=yes  8= I do not know/remember | | | |  |
|  | | M2 | | If yes, the last time you had this symptom, how many days did it last? | | ________ | | | |
|  | N1 | | Chest pain or tightness of the chest | | 0=no 1=yes  8= I do not know/remember | | | |  |
|  | | N2 | | If yes, the last time you had this symptom, how many days did it last? | | ________ | | | |
|  | O1 | | Muscle | | 0=no 1=yes  8= I do not know/remember | | | |  |
|  | | O2 | | If yes, the last time you had this symptom, how many days did it last? | | ________ | | | |
|  | P1 | | joint pain | | 0=no 1=yes  8= I do not know/remember | | | |  |
|  | | P2 | | If yes, the last time you had this symptom, how many days did it last? | | ________ | | | |
|  | Q1 | | Diarrhoea | | 0=no 1=yes  8= I do not know/remember | | | |  |
|  | | Q2 | | If yes, the last time you had this symptom, how many days did it last? | | _____________________ | | | |
| 6.2 | Did you seek care at a health facility for any of the mentioned symptoms?  *(If at least one answer to Q6.1 was “yes”)* | | | | | 0= no  1= yes, treated as an outpatient at a health facility  2= yes, was admitted to a hospital / health facility  8= I do not know/remember | | | |
|  | A | | Did you receive any drugs? | | 0=no 1=yes  8= I do not know/remember | | | |  |
|  | B | | Were you ever put on oxygen | | 0=no 1=yes  8= I do not know/remember | | | |  |
|  | C | | How many days were you admitted in hospital? | | 0=no 1=yes  8= I do not know/remember | | | |  |
| 6.3 | If you sought other care for any of the mentioned symptoms, please specify  ***(If at least one answer to Q6.1 was “yes”)*** | | | | | __________________________ | | | |
|  |  | | | | | | | | |
| 6.4 | **Did you experience any of the following symptoms since April 2020 up until now (excluding the past 2 weeks)?** | | | | | | | | |
|  | A1 | | Severe or unusual Headache | | 0=no 1=yes  8= I do not know/remember | | | |  |
|  | | A2 | | If yes, the last time you had this symptom, how many days did it last? | | ________ | | | |
|  | B1 | | Feeling more tired than usual | | 0=no 1=yes  8= I do not know/remember | | | |  |
|  | | B2 | | If yes, the last time you had this symptom, how many days did it last? | | ________ | | | |
|  | C1 | | Fever (feeling extremely hot) | | 0=no 1=yes  8= I do not know/remember | | | |  |
|  | | C2 | | If yes, the last time you had this symptom, how many days did it last? | | ________ | | | |
|  | D1 | | Chills (feeling extremely cold /shivering) | | 0=no 1=yes  8= I do not know/remember | | | |  |
|  | | D2 | | If yes, the last time you had this symptom, how many days did it last? | | ________ | | | |
|  | E1 | | Runny/blocked nose | | 0=no 1=yes  8= I do not know/remember | | | |  |
|  | | E2 | | If yes, the last time you had this symptom, how many days did it last? | | ________ | | | |
|  | F1 | | Loss of smell | | 0=no 1=yes  8= I do not know/remember | | | |  |
|  | | F2 | | If yes, the last time you had this symptom, how many days did it last? | | ________ | | | |
|  | G1 | | Shortness of breath or difficulty breathing | | 0=no 1=yes  8= I do not know/remember | | | |  |
|  | | G2 | | If yes, the last time you had this symptom, how many days did it last? | | ________ | | | |
|  | H1 | | wheezing | | 0=no 1=yes  8= I do not know/remember | | | |  |
|  | | H2 | | If yes, the last time you had this symptom, how many days did it last? | | ________ | | | |
|  | I1 | | New, persistent cough | | 0=no  1= yes non-productive  2=yes-productive  3=yes with blood  8=unknown | | | |  |
|  | | I2 | | If yes, the last time you had this symptom, how many days did it last? | | ________ | | | |
|  | J1 | | Nausea and/or vomiting | | 0=no 1=yes  8= I do not know/remember | | | |  |
|  | | J2 | | If yes, the last time you had this symptom, how many days did it last? | | ________ | | | |
|  | K1 | | Loss of appetite | | 0=no 1=yes  8= I do not know/remember | | | |  |
|  | | K2 | | If yes, the last time you had this symptom, how many days did it last? | | ________ | | | |
|  | L1 | | Loss of taste | | 0=no 1=yes  8= I do not know/remember | | | |  |
|  | | L2 | | If yes, the last time you had this symptom, how many days did it last? | | ________ | | | |
|  | M1 | | Sore throat | | 0=no 1=yes  8= I do not know/remember | | | |  |
|  | | M2 | | If yes, the last time you had this symptom, how many days did it last? | | ________ | | | |
|  | N1 | | Chest pain or tightness of the chest | | 0=no 1=yes  8= I do not know/remember | | | |  |
|  | | N2 | | If yes, the last time you had this symptom, how many days did it last? | | ________ | | | |
|  | O1 | | Muscle/ joint pain | | 0=no 1=yes  8= I do not know/remember | | | |  |
|  | | O2 | | If yes, the last time you had this symptom, how many days did it last? | | ________ | | | |
|  | P1 | | Diarrhoea | | 0=no 1=yes  8= I do not know/remember | | | |  |
|  | | P2 | | If yes, the last time you had this symptom, how many days did it last? | | _____________________ | | | |
| 6.5 | Did you seek care at a health facility for any of the mentioned symptoms?  *(If at least one answer to Q6.4 was “yes”)* | | | | | 0= no  1= yes, treated as an outpatient at a health facility  2= yes, was admitted to a hospital / health facility  8= I do not know/remember | | | |
|  | A | | Did you receive any drugs? | | 0=no 1=yes  8= I do not know/remember | | | |  |
|  | B | | Were you ever put on oxygen | | 0=no 1=yes  8= I do not know/remember | | | |  |
|  | C | | How many days were you admitted in hospital? | | 0=no 1=yes  8= I do not know/remember | | | |  |
| 6.6 | If you sought other care for any of the mentioned symptoms, please specify.  ***(If at least one answer to Q6.4 was “yes”)*** | | | | | __________________________ | | | |
|  |  | | | | |  | | | |
| 6.7 | **Did you experience any of the following symptoms since our last visit?**  ***(only relevant for subsequent visits)*** | | | | | | | | |
|  | A1 | | Severe or unusual Headache | | 0=no 1=yes  8= I do not know/remember | | | |  |
|  | | A2 | | If yes, the last time you had this symptom, how many days did it last? | | ________ | | | |
|  | B1 | | Feeling more tired than usual | | 0=no 1=yes  8= I do not know/remember | | | |  |
|  | | B2 | | If yes, the last time you had this symptom, how many days did it last? | | ________ | | | |
|  | C1 | | Fever (feeling hot) | | 0=no 1=yes  8= I do not know/remember | | | |  |
|  | | C2 | | If yes, the last time you had this symptom, how many days did it last? | | ________ | | | |
|  | D1 | | Chills (feeling cold) | | 0=no 1=yes  8= I do not know/remember | | | |  |
|  | | D2 | | If yes, the last time you had this symptom, how many days did it last? | | ________ | | | |
|  | E1 | | Runny/blocked nose | | 0=no 1=yes  8= I do not know/remember | | | |  |
|  | | E2 | | If yes, the last time you had this symptom, how many days did it last? | | ________ | | | |
|  | F1 | | Loss of smell | | 0=no 1=yes  8= I do not know/remember | | | |  |
|  | | F2 | | If yes, the last time you had this symptom, how many days did it last? | | ________ | | | |
|  | G1 | | Shortness of breath or difficulty breathing | | 0=no 1=yes  8= I do not know/remember | | | |  |
|  | | G2 | | If yes, the last time you had this symptom, how many days did it last? | | ________ | | | |
|  | H1 | | wheezing | | 0=no 1=yes  8= I do not know/remember | | | |  |
|  | | H2 | | If yes, the last time you had this symptom, how many days did it last? | | ________ | | | |
|  | I1 | | New, persistent cough | | 0=no  1= yes non-productive  2=yes productive  3=yes with blood  8=unknown | | | |  |
|  | | I2 | | If yes, the last time you had this symptom, how many days did it last? | | ________ | | | |
|  | J1 | | Nausea and/or vomiting | | 0=no 1=yes  8= I do not know/remember | | | |  |
|  | | J2 | | If yes, the last time you had this symptom, how many days did it last? | | ________ | | | |
|  | K1 | | Loss of appetite | | 0=no 1=yes  8= I do not know/remember | | | |  |
|  | | K2 | | If yes, the last time you had this symptom, how many days did it last? | | ________ | | | |
|  | L1 | | Loss of taste | | 0=no 1=yes  8= I do not know/remember | | | |  |
|  | | L2 | | If yes, the last time you had this symptom, how many days did it last? | | ________ | | | |
|  | M1 | | Sore throat | | 0=no 1=yes  8= I do not know/remember | | | |  |
|  | | M2 | | If yes, the last time you had this symptom, how many days did it last? | | ________ | | | |
|  | N1 | | Chest pain or tightness of the chest | | 0=no 1=yes  8= I do not know/remember | | | |  |
|  | | N2 | | If yes, the last time you had this symptom, how many days did it last? | | ________ | | | |
|  | O1 | | Muscle/ joint pain | | 0=no 1=yes  8= I do not know/remember | | | |  |
|  | | O2 | | If yes, the last time you had this symptom, how many days did it last? | | ________ | | | |
|  | P1 | | Diarrhoea | | 0=no 1=yes  8= I do not know/remember | | | |  |
|  | | P2 | | If yes, the last time you had this symptom, how many days did it last? | | _____________________ | | | |
| 6.8 | Did you seek care at a health facility for any of the mentioned symptoms?  *(If at least one answer to Q6.7 was “yes”)* | | | | | 0= no  1= yes, treated as an outpatient at a health facility  2=yes, was admitted to a hospital / health facility  8= I do not know/remember | | | |
|  | A | | Did you receive any drugs? | | 0=no 1=yes  8= I do not know/remember | | | |  |
|  | B | | Were you ever put on oxygen | | 0=no 1=yes  8= I do not know/remember | | | |  |
|  | C | | How many days were you admitted in hospital? | | 0=no 1=yes  8= I do not know/remember | | | |  |
| 6.9 | If you sought other care for any of the mentioned symptoms, please specify.  *(If at least one answer to Q6.6 was “yes”)* | | | | | __________________________ | | | |
|  |  | | | | |  | | | |
| **SECTION 7: COVID-19 VACCINE** | | | | | | | | | |
| 7.1 | Have you been offered a vaccine against COVID-19? | | | | | 0=no, not offered  1=yes, accepted and have had it  2=yes, accepted and have not yet had it  3=yes, refused | | | |
| 7.2 | Reason offered vaccine.  *(If answer to Q7.1 was not “No, not offered”)* | | | | | 1= Health Care worker  2=Age  3=Health condition  4=Other | | | |
| 7.3 | If there was another reason you were offered a vaccine, please specify.  (*If answer to Q7.2 was “Other”)* | | | | | __________________________ | | | |
| 7.4 | Reason refused vaccine:  (*If answer to Q7.1 was “Yes, refused”)* | | | | | ____________________________ | | | |
| 7.5.1 | How many vaccine doses have you received?  *(If answer to Q7.1 was “Yes, accepted and have had it”)* | | | | | ________________________ | | | |
| 7.5.2 | Date of first dose  *(If answer to Q7.1 was “Yes, accepted and have had it”)* | | | | | __________________________ | | | |
| 7.5.3 | Date of 2^nd^ Dose (if had):  *(If answer to Q7.1 was “Yes, accepted and have had it”)* | | | | | __________________________ | | | |
| 7.5.4 | Vaccine type received (if known)  *(If answer to Q7.1 was “Yes, accepted and have had it”)* | | | | | __________________________ | | | |
| 7.5.5 | Date due to receive vaccine:  *(If answer to Q7.1 was “Yes, accepted and have not yet had) it”.* | | | | | __________________________ | | | |
| 7.6 | Documentation regarding vaccination seen.  *(Question for interviewer)* | | | | | 0=no documentation seen  1=yes, official vaccine record i.e. health passport or vaccine card  2=yes, vaccine appointment letter | | | |
|  | | | | | | | | | |
| **SECTION 8: FOR FIELD WORKERS** | | | | | | | | | |
|  | Staff code | | | | |  | | | |
|  | Interview date | | | | |  | | | |

Table S2: COVSERO Household Questionnaire

| **SECTION 1. IDENTIFICATION** | | | | |  |
| --- | --- | --- | --- | --- | --- |
| 1.1 | Study site | | | **1**= Karonga | |
|  |  | | | **2**=Lilongwe | |
| 1.2 | Study ID | | | Study ID | |
|  |  |  |  | barcode | |
|  | (CRS) Household number | | | _____________________ | |
|  | Household GPS coordinates | | | ____________________ | |
|  |  |  |  |  |  |
| 1.3 | Contact number | | | Contact number barcode | |
| **SECTION 2: SOCIO-ECONOMIC INDICATORS** | | | | |  |
| 2.1 | Occupation of head of the household | | |  | |
| 2.2 | Total household monthly income | | | __________(3K- 8M Kwacha) | |
| 2.3 | How many people live in your household? | | | Number of people (1-20) | |
|  | List of the household occupants  ***(if answer to 2.3 is >1)***  ***These 3 variables should be entered for each household member)*** | | | ________(initials) | |
|  |  |  |  | ________age (0-110 yrs.) | |
|  |  |  |  | Relationship to head of house | |
| 2.4 | How many (sleeping) rooms does your house have? (excluding kitchen, bathroom or outside rooms unless people live there) | | | Number of rooms (1-10)  **Nambala ya zipinda (1-10)** | |
| 2.6 | Does your home have glass windows | | | 0= No 1=Yes | |
| 2.7 | Main source of water for hand washing | | | 1= Tap to house  2= Shared communal Tap  3= Bore hole  4= Covered well  5= Open well  6=Lake/river | |
| 2.8 | Does anyone in the household possess the following? | | | | |
|  | A | working watch or clock | | 0= No 1=Yes | |
|  | B | Working radio | | 0= No 1=Yes | |
|  | C | bank account (or bank book) | | 0= No 1=Yes | |
|  | D | Charcoal iron | | 0= No 1=Yes | |
|  | E | working sewing machine | | 0= No 1=Yes | |
|  | F | Mobile phone | | 0= No 1=Yes | |
|  | G | Mosquito net (number) | | 0= No 1=Yes | |
|  | H | Number of mosquito nets in the household | | 0= No 1=Yes | |
|  | I | Mattress | | 0= No 1=Yes | |
|  | J | Bed | | 0= No 1=Yes | |
|  | K | Bicycle | | 0= No 1=Yes | |
|  | L | Canoe | | 0= No 1=Yes | |
|  | M | Oxcart | | 0= No 1=Yes | |
|  | N | Motorbike | | 0= No 1=Yes | |
|  | O | Car | | 0= No 1=Yes | |
| 2.9 | Does anyone in the household possess something that requires electricity? | | | 0= No 1=Yes | |
| 2.91 | If yes, any of the following?  **(Circle for each item owned by the household. Only count if working)** | | | | |
|  | A | | Tape/CD player | 0= No 1=Yes | |
|  | B | | Fan | 0= No 1=Yes | |
|  | C | | Electric iron | 0= No 1=Yes | |
|  | D | | TV | 0= No 1=Yes | |
|  | E | | Fridge | 0= No 1=Yes | |
|  | F | | Other | 0= No 1=Yes | |
|  |  | |  |  | |

Table S3: In-house multiplex respiratory PCR design

| Target | Reference |
| --- | --- |
| Adenovirus | Brittain-Long et al., 2008 |
| Bocavirus | Lu et al., 2006 |
| Enterovirus | [46] |
| Influenza A | [47] |
| Influenza B | [47] |
| Influenza C | Howard et al., 2017 |
| Metapneumovirus | [48] |
| Parainfluenza 1 | [47] |
| Parainfluenza 2 | [47] |
| Parainfluenza 3 | [47] |
| Parainfluenza 4 | [47] |
| Parechovirus | [46] |
| Rhinovirus | [49] |
| RSV | [48] |

Table S4: Scoring components

| Score | **NPI Score** | **Asset Score** | **Behaviour Score** |
| --- | --- | --- | --- |
| Components | Face mask | Watch or clock | Attended funeral in past week |
|  | Avoiding shaking hands | Radio | Attended place of work in past week |
|  | Avoiding touching own face | Charcoal iron | Attended religious meeting in past week |
|  | Maintaining 1 metre social distancing | Sewing machine | Used public transport in past week |
|  | Increased hand washing | Mobile phone | Visited healthcare facility in past week |
|  | Avoiding people with cough or fever | Mosquito net | Visited markets/shops in past week |
|  | Staying at home | Mattress | Visited bars/clubs in past week |
|  | Avoiding crowds | Bed | Visited hairdresser/beauty salon in past week |
|  | Avoiding hospitals | Bicycle | Attended wedding/ engagement/ bridal shower in past week |
|  |  | Canoe | Attended demonstration in past week |
|  |  | Oxcart | Travel outside of residence in past week |
|  |  | Electrical household items | Travel outside of local district in past week |
|  |  | Car |  |
|  |  | Motorcycle |  |
|  |  | Tape/CD player |  |
|  |  | Fan |  |
|  |  | Electric iron |  |
|  |  | Television |  |
|  |  | Refrigerator |  |

Table S5: Forward model selection process

| **Outcome variable** | **Step** | **Model** | **AIC** | **ΔAIC** | **p-value** |
| --- | --- | --- | --- | --- | --- |
| Respiratory virus PCR positive (including SARS-CoV-2) | 1 | 1 + site + age + season_year | 826.827 | NA | NA |
|  | 2 | **1 + site + age + season_year + work** | **824.812** | **-2.015** | **0.0451** |
|  | 3 | 1 + site + age + season_year + work + symptomatic_under5 | 823.656 | -1.156 | 0.0757 |
| Respiratory virus PCR positive (excluding SARS-CoV-2) | 1 | 1 + site + age + season_year | 472.598 | NA | NA |
|  | 2 | **1 + site + age + season_year + occpub** | **401.956** | **-70.642** | **1.55E-17** |
|  | 3 | 1 + site + age + season_year + occpub + numb_in_house | 407.679 | 5.722 | 1 |

Mixed effect logistic regression models were used throughout, with a nested random effect term of participant within household. Initial model contained *a priori* selected variables and intercept only. Candidate variables were considered systematically, the model with lowest AIC was selected, and model complexity increased only if a likelihood ratio test p-value<0.05. p-values refer to the significance of a likelihood ratio test between that model and the model from the previous step. Bold denotes final model selection.

Variables chosen *a priori*: location (site); age group (age); season of study (season_year)

Candidate variables: attendance at place of work in past week (work), public-facing occupation (occpub); presence of symptomatic children aged <5years in household (symptomatic_under5); presence of symptomatic children aged <15years in household (symptomatic_under15); presence of symptomatic person in household (hhid_symptomatic); number of people in household (numb_in_house); sex (sex); attendance at funeral in past week (funeral); used public transport in past week (transport); NPI Score (precaution_score); Exposure risk behaviour score (behaviour_score); 1 denotes intercept term.

Table S6. Mixed-effects logistic regression model summaries

**a)** **Outcome: Respiratory virus PCR positive (including SARS_CoV-2)**

| **Fixed effect coefficients** | **Estimate** | **Std. Error** | **z value** | **aOR** | **2.5% CI** | **97.5% CI** | **p value** |
| --- | --- | --- | --- | --- | --- | --- | --- |
| **(Intercept)** | -3.71 | 0.44 | -8.41 | 0.02 | 0.01 | 0.06 | 4.21E-17 |
| **Site: Karonga** | 0.95 | 0.33 | 2.87 | 2.58 | 1.35 | 4.91 | 0.004 |
| **Season: Cool/dry 2021** | -0.16 | 0.40 | -0.40 | 0.85 | 0.39 | 1.85 | 0.686 |
| **Season: Hot/dry 2021** | -0.74 | 0.42 | -1.77 | 0.48 | 0.21 | 1.08 | 0.077 |
| **Season: Hot/rainy 2021-22** | -1.23 | 0.44 | -2.83 | 0.29 | 0.12 | 0.69 | 0.004 |
| **Age group: 40-59** | -0.30 | 0.34 | -0.88 | 0.74 | 0.38 | 1.45 | 0.381 |
| **Age group: >=60** | 0.48 | 0.45 | 1.07 | 1.61 | 0.67 | 3.86 | 0.283 |
| **Attended place of work in past two weeks: Yes** | 0.45 | 0.30 | 1.47 | 1.56 | 0.86 | 2.83 | 0.140 |

Abbreviations: Std. Error, standard error; aOR, adjusted odds ratio; CI, confidence interval

**b) Outcome: Respiratory virus PCR positive (excluding SARS_CoV-2)**

| **Fixed effect coefficients** | **Estimate** | **Std. Error** | **z value** | **aOR** | **2.5% CI** | **97.5% CI** | **p value** |
| --- | --- | --- | --- | --- | --- | --- | --- |
| **(Intercept)** | -2.91 | 0.40 | -7.36 | 0.05 | 0.02 | 0.12 | 1.87E-13 |
| **Site: Karonga** | 0.61 | 0.29 | 2.11 | 1.84 | 1.04 | 3.24 | 0.035 |
| **Season: Cool/dry 2021** | -0.67 | 0.39 | -1.74 | 0.51 | 0.24 | 1.09 | 0.082 |
| **Season: Hot/dry 2021** | -0.77 | 0.40 | -1.92 | 0.46 | 0.21 | 1.02 | 0.055 |
| **Season: Hot/rainy 2021-22** | -1.33 | 0.44 | -3.03 | 0.26 | 0.11 | 0.62 | 0.002 |
| **Age group: 40-59** | -0.30 | 0.32 | -0.95 | 0.74 | 0.40 | 1.38 | 0.344 |
| **Age group: >=60** | -0.05 | 0.45 | -0.11 | 0.95 | 0.39 | 2.32 | 0.915 |
| **Public-facing occupation: Yes** | 0.18 | 0.29 | 0.61 | 1.19 | 0.67 | 2.11 | 0.541 |

Abbreviations: Std. Error, standard error; aOR, adjusted odds ratio; CI, confidence interval

Table S7: Respiratory Viruses detected, stratified by location, season, sex, and age.

| **Virus PCR Positivity** | | **Site** | | **Season & Year** | | | | **Sex** | | **Age Group (years)** | | |
| --- | --- | --- | --- | --- | --- | --- | --- | --- | --- | --- | --- | --- |
|  | **n (%)** | **Lilongwe** | **Karonga** | **Hot/rainy**  **2020-21** | **Cool/dry 2021** | **Hot/dry 2021** | **Hot/rainy**  **2021-22** | **Female** | **Male** | **15-39** | **40-59** | **≥60** |
| **Samples tested** | 1626 | 912 | 714 | 168 | 526 | 442 | 490 | 972 | 654 | 920 | 529 | 177 |
| **All respiratory viruses** | 123 (7.6) | 45 (4.9) | 78 (10.9) | 20 (11.9) | 50 (9.5) | 29 (6.6) | 24 (4.9) | 72 (7.4) | 51 (7.8) | 73 (7.9) | 32 (6.0) | 18 (10.2) |
| **All respiratory viruses (excluding SARS-CoV-2)** | 55 (3.3) | 23 (2.5) | 32 (4.5) | 12 (7.1) | 18 (3.4) | 15 (3.4) | 10 (2.0) | 34 (3.5) | 21 (3.2) | 34 (3.7) | 21 (3.2) | 6 (3.4) |
| **SARS-CoV-2** | 71 (4.4) | 22 (2.4) | 49 (6.9) | 9 (5.4) | 34 (6.5) | 14 (3.2) | 14 (2.9) | 40 (4.1) | 31 (4.7) | 40 (4.3) | 18 (3.4) | 13 (7.3) |
| **Rhinovirus** | 33 (2.0) | 14 (1.5) | 19 (2.7) | 7 (4.2) | 8 (1.5) | 10 (2.3) | 8 (1.6) | 20 (2.1) | 13 (2.0) | 20 (2.2) | 9 (1.7) | 4 (2.3) |
| **Adenovirus** | 9 (0.6) | 2 (0.2) | 7 (1.0) | 3 (1.8) | 4 (0.8) | 1 (0.2) | 1 (0.2) | 6 (0.6) | 3 (0.5) | 7 (0.8) | 2 (0.4) | 0 (0.0) |
| **Bocavirus** | 4 (0.3) | 2 (0.2) | 2 (0.3) | 1 (0.6) | 1 (0.2) | 2 (0.5) | 0 (0.0) | 3 (0.3) | 1 (0.2) | 1 (0.1) | 3 (0.6) | 0 (0.0) |
| **Parainfluenza viruses** | 2 (0.12) | 0 (0.0) | 2 (0.3) | 0 (0.0) | 2 (0.4) | 0 (0.0) | 0 (0.0) | 1 (0.1) | 1 (0.2) | 1 (0.1) | 1 (0.2) | 0 (0.0) |
| **Influenza C virus** | 2 (0.12) | 2 (0.2) | 0 (0.0) | 0 (0.0) | 0 (0.0) | 2 (0.5) | 0 (0.0) | 2 (0.2) | 0 (0.0) | 2 (0.2) | 0 (0.0) | 0 (0.0) |
| **RSV** | 2 (0.12) | 1 (0.1) | 1 (0.1) | 0 (0.0) | 1 (0.2) | 0 (0.0) | 1 (0.2) | 1 (0.1) | 1 (0.2) | 2 (0.2) | 0 (0.0) | 0 (0.0) |
| **Enterovirus** | 1 (0.06) | 0 (0.0) | 1 (0.1) | 0 (0.0) | 1 (0.2) | 0 (0.0) | 0 (0.0) | 1 (0.1) | 0 (0.0) | 0 (0.0) | 0 (0.0) | 1 (0.6) |
| **Human metapneumovirus** | 1 (0.06) | 1 (0.1) | 0 (0.0) | 0 (0.0) | 1 (0.2) | 0 (0.0) | 0 (0.0) | 0 (0.0) | 1 (0.2) | 0 (0.0) | 0 (0.0) | 1 (0.6) |
| **Parechovirus** | 1 (0.06) | 0 (0.0) | 1 (0.1) | 1 (0.6) | 0 (0.0) | 0 (0.0) | 0 (0.0) | 0 (0.0) | 1 (0.2) | 1 (0.1) | 0 (0.0) | 0 (0.0) |
| **Influenza B virus** | 1 (0.06) | 1 (0.1) | 0 (0.0) | 0 (0.0) | 1 (0.2) | 0 (0.0) | 0 (0.0) | 1 (0.1) | 0 (0.0) | 1 (0.1) | 0 (0.0) | 0 (0.0) |
| **Influenza A virus** | 0 (0.0) | 0 (0.0) | 0 (0.0) | 0 (0.0) | 0 (0.0) | 0 (0.0) | 0 (0.0) | 0 (0.0) | 0 (0.0) | 0 (0.0) | 0 (0.0) | 0 (0.0) |

Abbreviations: PCR, polymerase chain reaction; SARS-CoV-2, severe acute respiratory syndrome coronavirus 2; RSV, respiratory syncytial virus

Table S8. Symptoms associated with recurrent SARS-CoV-2 detection in an individual

| **visit** | **Fever** | **New, persistent cough** | **Severe or unusual headache** | **Fatigue** | **Chills** | **Runny or blocked nose** | **Loss of smell** | **Shortness of breath** | **Wheeze** | **Nausea and/or vomiting** | **Low appetite** | **Loss of taste** | **Sore throat** | **Chest pain** | **Myalgia** | **Joint pain** | **Diarrhoea** |
| --- | --- | --- | --- | --- | --- | --- | --- | --- | --- | --- | --- | --- | --- | --- | --- | --- | --- |
| Survey 1 | Yes | Yes | No | Yes | Yes | Yes | No | Yes | No | No | No | No | No | Yes | Yes | Yes | Yes |
| Survey 2 | Yes | No | No | Yes | No | Yes | No | No | Yes | No | No | No | No | No | Yes | Yes | Yes |
| Survey 3 | No | No | No | No | No | Yes | No | No | No | No | No | No | No | No | No | No | No |

Table S9. Symptoms associated with respiratory virus positivity (including SARS-CoV-2); univariable and multivariable logistic regression analysis.

| **Symptom** |  | **PCR-positivity for >1 respiratory virus** | | **Univariable**  **Analysis** | | **Multivariable**  **Analysis** | |
| --- | --- | --- | --- | --- | --- | --- | --- |
|  |  | **Negative** | **Positive** | **OR**  **(95% CI)** | **p value** | **aOR**  **(95% CI)** | **p value** |
| **Fever** | No | 1402 (92.8) | 109 (7.2) | - |  | - |  |
|  | Yes | 99 (87.6) | 14 (12.4) | 1.82  (0.97-3.19) | 0.048 | 0.85  (0.36-1.88) | 0.702 |
| **New, persistent cough** | No | 1387 (92.8) | 108 (7.2) | - |  | - |  |
|  | Yes | 113 (88.3) | 15 (11.7) | 1.70  (0.93-2.94) | 0.068 | 1.20  (0.61-2.28) | 0.581 |
| **Severe or unusual headache** | No | 1277 (92.4) | 105 (7.6) | - |  | - |  |
|  | Yes | 226 (92.6) | 18 (7.4) | 0.97  (0.56-1.59) | 0.904 | 0.57  (0.28-1.09) | 0.099 |
| **Fatigue** | No | 1412 (92.9) | 108 (7.1) | - |  | - |  |
|  | Yes | 89 (85.6) | 15 (14.4) | 2.20  (1.19-3.83)) | 0.008 | 1.60  (0.73-3.34) | 0.220 |
| **Chills** | No | 1412 (93.1) | 104 (6.9) | - |  | - |  |
|  | Yes | 91 (82.7) | 19 (17.3) | 2.83  (1.62-4.74) | <0.001 | 2.00  (0.94-4.10) | 0.062 |
| **Runny/blocked nose** | No | 1355 (93.9) | 88 (6.1) | - |  | - |  |
|  | Yes | 148 (80.9) | 35 (19.1) | 3.64  (2.35-5.54) | <0.001 | 3.41  (1.90-6.19) | <0.001 |
| **Loss of smell** | No | 1457 (92.8) | 113 (7.2) | - |  | - |  |
|  | Yes | 45 (83.3) | 9 (16.7) | 2.58  (1.16-5.17) | 0.012 | 1.03  (0.39-2.52) | 0.944 |
| **Shortness of breath** | No | 1468 (92.7) | 115 (7.3) | - |  | - |  |
|  | Yes | 33 (80.5) | 8 (19.5) | 3.09  (1.30-6.54) | 0.005 | 1.25  (0.45-3.09) | 0.643 |
| **Wheeze** | No | 1482 (92.7) | 116 (7.3) | - |  | - |  |
|  | Yes | 17 (70.8) | 7 (29.2) | 5.26  (2.00-12.46) | <0.001 | 3.77  (1.24-10.69) | 0.015 |
| **Nausea and/or vomiting** | No | 1438 (92.5) | 117 (7.5) | - |  | - |  |
|  | Yes | 65 (91.5) | 6 (8.5) | 1.13  (0.43-2.47) | 0.773 | 0.51  (0.16-1.36) | 0.218 |
| **Low appetite** | No | 1453 (92.8) | 113 (7.2) | - |  | - |  |
|  | Yes | 49 (83.1) | 10 (16.9) | 2.62  (1.23-5.11) | 0.007 | 1.67  (0.53-4.77) | 0.356 |
| **Loss of taste** | No | 1454 (92.7) | 114 (7.3) | - |  | - |  |
|  | Yes | 47 (83.9) | 9 (16.1) | 2.44  (1.10-4.88) | 0.018 | 0.68  (0.20-2.14) | 0.527 |
| **Sore throat** | No | 1478 (92.7) | 117 (7.3) | - |  | - |  |
|  | Yes | 24 (80.0) | 6 (20.0) | 3.16  (1.15-7.41) | 0.014 | 1.60  (0.49-4.36) | 0.395 |
| **Chest pain** | No | 1458 (92.7) | 115 (7.3) | - |  | - |  |
|  | Yes | 43 (84.3) | 8 (15.7) | 2.36  (1.01-4.88) | 0.031 | 1.06  (0.37-2.69) | 0.913 |
| **Myalgia** | No | 1423 (92.9) | 108 (7.1) | - |  | - |  |
|  | Yes | 79 (84.0) | 15 (16.0) | 2.50  (1.35-4.38) | 0.002 | 1.36  (0.62-2.82) | 0.426 |
| **Joint pain** | No | 1416 (92.8) | 110 (7.2) | - |  | - |  |
|  | Yes | 86 (86.9) | 13 (13.1) | 1.95  (1.01-3.48) | 0.034 | 1.34  (0.59-2.85) | 0.465 |
| **Diarrhoea** | No | 1428 (92.7) | 113 (7.3) | - |  | - |  |
|  | Yes | 75 (88.2) | 10 (11.8) | 1.68  (0.80-3.20) | 0.137 | 1.11  (0.46-2.44) | 0.807 |
| **Any respiratory virus symptoms** | No | 919 (93.8) | 61 (6.2) | - |  | - |  |
|  | Yes | 579 (90.3) | 62 (9.7) | 1.61  (1.12-2.33) | 0.011 | 0.75  (0.41-1.33) | 0.332 |

Abbreviations: PCR, polymerase chain reaction; OR, odds ratio; CI, confidence interval; aOR, adjusted

odds ratio

Table S10: Univariable logistic regression analysis of risk factors for respiratory virus positivity including SARS-CoV-2

| Risk Factors |  | Respiratory Virus PCR Negative (including SARS-CoV-2) | Respiratory Virus PCR Positive (including SARS-CoV-2) | OR (univariable) |
| --- | --- | --- | --- | --- |
| Site | Lilongwe | 867 (95.1) | 45 (4.9) | - |
|  | Karonga | 636 (89.1) | 78 (10.9) | 2.36 (1.62-3.48, p<0.01) |
| Season & Year | Hot/rainy 2020-21 | 148 (88.1) | 20 (11.9) | - |
|  | Cool/dry 2021 | 476 (90.5) | 50 (9.5) | 0.78 (0.45-1.37, p=0.37) |
|  | Hot/dry 2021 | 413 (93.4) | 29 (6.6) | 0.52 (0.29-0.96, p=0.03) |
|  | Hot/rainy 2021-22 | 466 (95.1) | 24 (4.9) | 0.38 (0.20-0.72, p<0.01) |
| Sex | Female | 900 (92.6) | 72 (7.4) | - |
|  | Male | 603 (92.2) | 51 (7.8) | 1.06 (0.72-1.53, p=0.77) |
| Age Group (years) | 15-39 | 847 (92.1) | 73 (7.9) | - |
|  | 40-59 | 497 (94.0) | 32 (6.0) | 0.75 (0.48-1.14, p=0.18) |
|  | >=60 | 159 (89.8) | 18 (10.2) | 1.31 (0.74-2.21, p=0.32) |
| COVID-19 Vaccination Status | Not Vaccinated | 1051 (91.9) | 93 (8.1) | - |
|  | Vaccinated | 398 (93.9) | 26 (6.1) | 0.74 (0.46-1.14, p=0.19) |
| Number of comorbidities | 0 | 1203 (92.3) | 100 (7.7) | - |
|  | 1 | 268 (92.4) | 22 (7.6) | 0.99 (0.60-1.57, p=0.96) |
|  | 2 | 32 (97.0) | 1 (3.0) | 0.38 (0.02-1.78, p=0.34) |
| Hypertension | No | 1318 (92.4) | 108 (7.6) | - |
|  | Yes | 185 (92.5) | 15 (7.5) | 0.99 (0.54-1.68, p=0.97) |
| Asthma | No | 1402 (92.4) | 115 (7.6) | - |
|  | Yes | 101 (92.7) | 8 (7.3) | 0.97 (0.42-1.92, p=0.93) |
| Chronic Lung Disease (excluding asthma) | No | 1498 (92.5) | 122 (7.5) | - |
|  | Yes | 5 (83.3) | 1 (16.7) | 2.46 (0.13-15.38, p=0.41) |
| Heart Disease | No | 1483 (92.5) | 121 (7.5) | - |
|  | Yes | 20 (90.9) | 2 (9.1) | 1.23 (0.19-4.26, p=0.79) |
| HIV | HIV Negative | 1376 (92.5) | 111 (7.5) | - |
|  | HIV Positive | 127 (91.4) | 12 (8.6) | 1.17 (0.60-2.10, p=0.62) |
| Highest level of education | Secondary or Higher | 871 (93.6) | 60 (6.4) | - |
|  | Primary or None | 632 (90.9) | 63 (9.1) | 1.45 (1.00-2.09, p=0.05) |
| Number in household | 1-2 | 89 (95.7) | 4 (4.3) | - |
|  | 3-5 | 744 (92.2) | 63 (7.8) | 1.88 (0.75-6.31, p=0.23) |
|  | >5 | 665 (92.2) | 56 (7.8) | 1.87 (0.75-6.29, p=0.24) |
| Child/children under 5 years in household | No | 163 (91.6) | 15 (8.4) | - |
|  | Yes | 129 (90.8) | 13 (9.2) | 1.10 (0.50-2.39, p=0.82) |
| Symptomatic child under 5 years in household | No | 1376 (92.8) | 106 (7.2) | - |
|  | Yes | 127 (88.2) | 17 (11.8) | 1.74 (0.98-2.92, p=0.05) |
| Symptomatic child under 15 years in household | No | 1079 (93.1) | 80 (6.9) | - |
|  | Yes | 424 (90.8) | 43 (9.2) | 1.37 (0.92-2.00, p=0.11) |
| Symptomatic person in household | No | 467 (95.1) | 24 (4.9) | - |
|  | Yes | 1034 (91.3) | 99 (8.7) | 1.86 (1.20-3.01, p=0.01) |
| Occupation Type | Regular wage/salary | 234 (92.9) | 18 (7.1) | - |
|  | Unwaged | 842 (91.4) | 79 (8.6) | 1.22 (0.73-2.14, p=0.46) |
|  | Irregular wage/piecework | 427 (94.3) | 26 (5.7) | 0.79 (0.43-1.50, p=0.46) |
| Outdoor Worker | No | 606 (92.9) | 46 (7.1) | - |
|  | Yes | 897 (92.1) | 77 (7.9) | 1.13 (0.78-1.66, p=0.53) |
| Public Facing Occupation | No | 683 (93.3) | 49 (6.7) | - |
|  | Yes | 818 (91.7) | 74 (8.3) | 1.26 (0.87-1.84, p=0.23) |
| Attended place of work in past week | No | 773 (94.2) | 48 (5.8) | - |
|  | Yes | 730 (90.7) | 75 (9.3) | 1.65 (1.14-2.42, p=0.01) |
| Attended religious meeting in past week | No | 766 (92.6) | 61 (7.4) | - |
|  | Yes | 737 (92.2) | 62 (7.8) | 1.06 (0.73-1.53, p=0.77) |
| Attended funeral in past week | No | 1096 (93.5) | 76 (6.5) | - |
|  | Yes | 407 (89.6) | 47 (10.4) | 1.67 (1.13-2.43, p=0.01) |
| Used public transport in past week | No | 938 (92.9) | 72 (7.1) | - |
|  | Yes | 565 (91.7) | 51 (8.3) | 1.18 (0.81-1.70, p=0.40) |
| Visited healthcare facility in past week | No | 1228 (92.3) | 103 (7.7) | - |
|  | Yes | 275 (93.2) | 20 (6.8) | 0.87 (0.51-1.39, p=0.57) |
| Visited markets/shops in past week | No | 414 (91.2) | 40 (8.8) | - |
|  | Yes | 1089 (92.9) | 83 (7.1) | 0.79 (0.54-1.18, p=0.24) |
| Visited hairdresser/beauty salon in past week | No | 1272 (92.4) | 104 (7.6) | - |
|  | Yes | 231 (92.4) | 19 (7.6) | 1.01 (0.59-1.64, p=0.98) |
| Visited bars/clubs in past week | No | 1324 (92.3) | 111 (7.7) | - |
|  | Yes | 179 (93.7) | 12 (6.3) | 0.80 (0.41-1.42, p=0.48) |
| Attended wedding/engagement/bridal shower in past week | No | 1398 (92.2) | 119 (7.8) | - |
|  | Yes | 105 (96.3) | 4 (3.7) | 0.45 (0.14-1.09, p=0.12) |
| Attended demonstration in past week | No | 1463 (92.3) | 122 (7.7) | - |
|  | Yes | 40 (97.6) | 1 (2.4) | 0.30 (0.02-1.40, p=0.24) |
| Travel outside of residence in past week | No | 826 (92.3) | 69 (7.7) | - |
|  | Yes | 677 (92.6) | 54 (7.4) | 0.95 (0.66-1.38, p=0.81) |
| Travel outside of local district in past week | No | 1329 (92.2) | 112 (7.8) | - |
|  | Yes | 174 (94.1) | 11 (5.9) | 0.75 (0.37-1.36, p=0.38) |
| Behaviour Score* | Mean (SD) | 3.5 (1.8) | 3.6 (1.7) | 1.03 (0.93-1.15, p=0.53) |
| Precaution Score* | Mean (SD) | 4.7 (2.7) | 4.6 (2.8) | - 1. 0.92-1.06, p=0.68) |

* For scoring components see [Table S2](#_Table_S2:_Scoring)

Table S11: Univariable logistic regression analysis of risk factors for respiratory virus positivity excluding SARS-CoV-2

| Risk Factors |  | Respiratory Virus PCR Negative (excluding SARS-CoV-2) | Respiratory Virus PCR Positive (excluding SARS-CoV-2) | OR (univariable) |
| --- | --- | --- | --- | --- |
| Site | Lilongwe | 889 (97.5) | 23 (2.5) | - |
|  | Karonga | 682 (95.5) | 32 (4.5) | 1.81 (1.06-3.16, p=0.03) |
| Season & Year | Hot/rainy 2020-21 | 156 (92.9) | 12 (7.1) | - |
|  | Cool/dry 2021 | 508 (96.6) | 18 (3.4) | 0.46 (0.22-1.00, p=0.04) |
|  | Hot/dry 2021 | 427 (96.6) | 15 (3.4) | 0.46 (0.21-1.02, p=0.05) |
|  | Hot/rainy 2021-22 | 480 (98.0) | 10 (2.0) | 0.27 (0.11-0.64, p<0.01) |
| Sex | Female | 938 (96.5) | 34 (3.5) | - |
|  | Male | 633 (96.8) | 21 (3.2) | 0.92 (0.52-1.58, p=0.75) |
| Age Group (years) | 15-39 | 886 (96.3) | 34 (3.7) | - |
|  | 40-59 | 514 (97.2) | 15 (2.8) | 0.76 (0.40-1.38, p=0.38) |
|  | >=60 | 171 (96.6) | 6 (3.4) | 0.91 (0.34-2.06, p=0.84) |
| COVID-19 Vaccination Status | Not Vaccinated | 1098 (96.0) | 46 (4.0) | - |
|  | Vaccinated | 415 (97.9) | 9 (2.1) | 0.52 (0.24-1.02, p=0.07) |
| Number of comorbidities | 0 | 1260 (96.7) | 43 (3.3) | - |
|  | 1 | 278 (95.9) | 12 (4.1) | 1.26 (0.63-2.35, p=0.48) |
|  | 2 | 33 (100.0) | 0 (0.0) | 0.00 (0.00-120227725.90, p=0.98) |
| Hypertension | No | 1378 (96.6) | 48 (3.4) | - |
|  | Yes | 193 (96.5) | 7 (3.5) | 1.04 (0.43-2.19, p=0.92) |
| Asthma | No | 1467 (96.7) | 50 (3.3) | - |
|  | Yes | 104 (95.4) | 5 (4.6) | 1.41 (0.48-3.29, p=0.47) |
| Chronic Lung Disease (excluding asthma) | No | 1566 (96.7) | 54 (3.3) | - |
|  | Yes | 5 (83.3) | 1 (16.7) | 5.80 (0.30-36.78, p=0.11) |
| Heart Disease | No | 1549 (96.6) | 55 (3.4) | - |
|  | Yes | 22 (100.0) | 0 (0.0) | 0.00 (NA-196121124817944.16, p=0.99) |
| HIV | HIV Negative | 1438 (96.7) | 49 (3.3) | - |
|  | HIV Positive | 133 (95.7) | 6 (4.3) | 1.32 (0.50-2.92, p=0.53) |
| Highest level of education | Secondary or Higher | 906 (97.3) | 25 (2.7) | - |
|  | Primary or None | 665 (95.7) | 30 (4.3) | 1.63 (0.95-2.83, p=0.07) |
| Number in household | 1-2 | 91 (97.8) | 2 (2.2) | - |
|  | 3-5 | 778 (96.4) | 29 (3.6) | 1.70 (0.50-10.60, p=0.47) |
|  | >5 | 697 (96.7) | 24 (3.3) | 1.57 (0.45-9.85, p=0.55) |
| Child/children under 5 years in household | No | 168 (94.4) | 10 (5.6) | - |
|  | Yes | 131 (92.3) | 11 (7.7) | 1.41 (0.58-3.48, p=0.45) |
| Symptomatic child under 5 years in household | No | 1436 (96.9) | 46 (3.1) | - |
|  | Yes | 135 (93.8) | 9 (6.2) | 2.08 (0.94-4.15, p=0.05) |
| Symptomatic child under 15 years in household | No | 1122 (96.8) | 37 (3.2) | - |
|  | Yes | 449 (96.1) | 18 (3.9) | 1.22 (0.67-2.13, p=0.50) |
| Symptomatic person in household | No | 478 (97.4) | 13 (2.6) | - |
|  | Yes | 1091 (96.3) | 42 (3.7) | 1.42 (0.77-2.77, p=0.28) |
| Occupation Type | Regular wage/salary | 243 (96.4) | 9 (3.6) | - |
|  | Unwaged | 889 (96.5) | 32 (3.5) | 0.97 (0.48-2.19, p=0.94) |
|  | Irregular wage/piecework | 439 (96.9) | 14 (3.1) | 0.86 (0.37-2.09, p=0.73) |
| Outdoor Worker | No | 631 (96.8) | 21 (3.2) | - |
|  | Yes | 940 (96.5) | 34 (3.5) | 1.09 (0.63-1.92, p=0.77) |
| Public Facing Occupation | No | 711 (97.1) | 21 (2.9) | - |
|  | Yes | 858 (96.2) | 34 (3.8) | 1.34 (0.78-2.37, p=0.30) |
| Attended place of work in past week | No | 801 (97.6) | 20 (2.4) | - |
|  | Yes | 770 (95.7) | 35 (4.3) | 1.82 (1.05-3.24, p=0.04) |
| Attended religious meeting in past week | No | 800 (96.7) | 27 (3.3) | - |
|  | Yes | 771 (96.5) | 28 (3.5) | 1.08 (0.63-1.85, p=0.79) |
| Attended funeral in past week | No | 1141 (97.4) | 31 (2.6) | - |
|  | Yes | 430 (94.7) | 24 (5.3) | 2.05 (1.18-3.53, p=0.01) |
| Used public transport in past week | No | 975 (96.5) | 35 (3.5) | - |
|  | Yes | 596 (96.8) | 20 (3.2) | 0.93 (0.53-1.62, p=0.81) |
| Visited healthcare facility in past week | No | 1283 (96.4) | 48 (3.6) | - |
|  | Yes | 288 (97.6) | 7 (2.4) | 0.65 (0.27-1.36, p=0.29) |
| Visited markets/shops in past week | No | 438 (96.5) | 16 (3.5) | - |
|  | Yes | 1133 (96.7) | 39 (3.3) | 0.94 (0.53-1.75, p=0.84) |
| Visited hairdresser/beauty salon in past week | No | 1331 (96.7) | 45 (3.3) | - |
|  | Yes | 240 (96.0) | 10 (4.0) | 1.23 (0.58-2.38, p=0.56) |
| Visited bars/clubs in past week | No | 1384 (96.4) | 51 (3.6) | - |
|  | Yes | 187 (97.9) | 4 (2.1) | 0.58 (0.17-1.44, p=0.30) |
| Attended wedding/engagement/bridal shower in past week | No | 1463 (96.4) | 54 (3.6) | - |
|  | Yes | 108 (99.1) | 1 (0.9) | 0.25 (0.01-1.16, p=0.17) |
| Attended demonstration in past week | No | 1530 (96.5) | 55 (3.5) | - |
|  | Yes | 41 (100.0) | 0 (0.0) | 0.00 (0.00-426867.61, p=0.98) |
| Travel outside of residence in past week | No | 864 (96.5) | 31 (3.5) | - |
|  | Yes | 707 (96.7) | 24 (3.3) | 0.95 (0.55-1.62, p=0.84) |
| Travel outside of local district in past week | No | 1390 (96.5) | 51 (3.5) | - |
|  | Yes | 181 (97.8) | 4 (2.2) | 0.60 (0.18-1.50, p=0.33) |
| Behaviour score* | Mean (SD) | 3.5 (1.8) | 3.6 (1.8) | 1.03 (0.88-1.20, p=0.70) |
| NPI Score* | Mean (SD) | 4.7 (2.7) | 5.1 (3.0) | 1.05 (0.95-1.16, p=0.32) |

* For scoring components see [Table S2](#_Table_S2:_Scoring)

Figure S1. Spatial analysis of virus positivity by location; a) any respiratory virus, b) a respiratory virus excluding SARS-CoV-2, c) rhinovirus, and d) adenovirus

a)


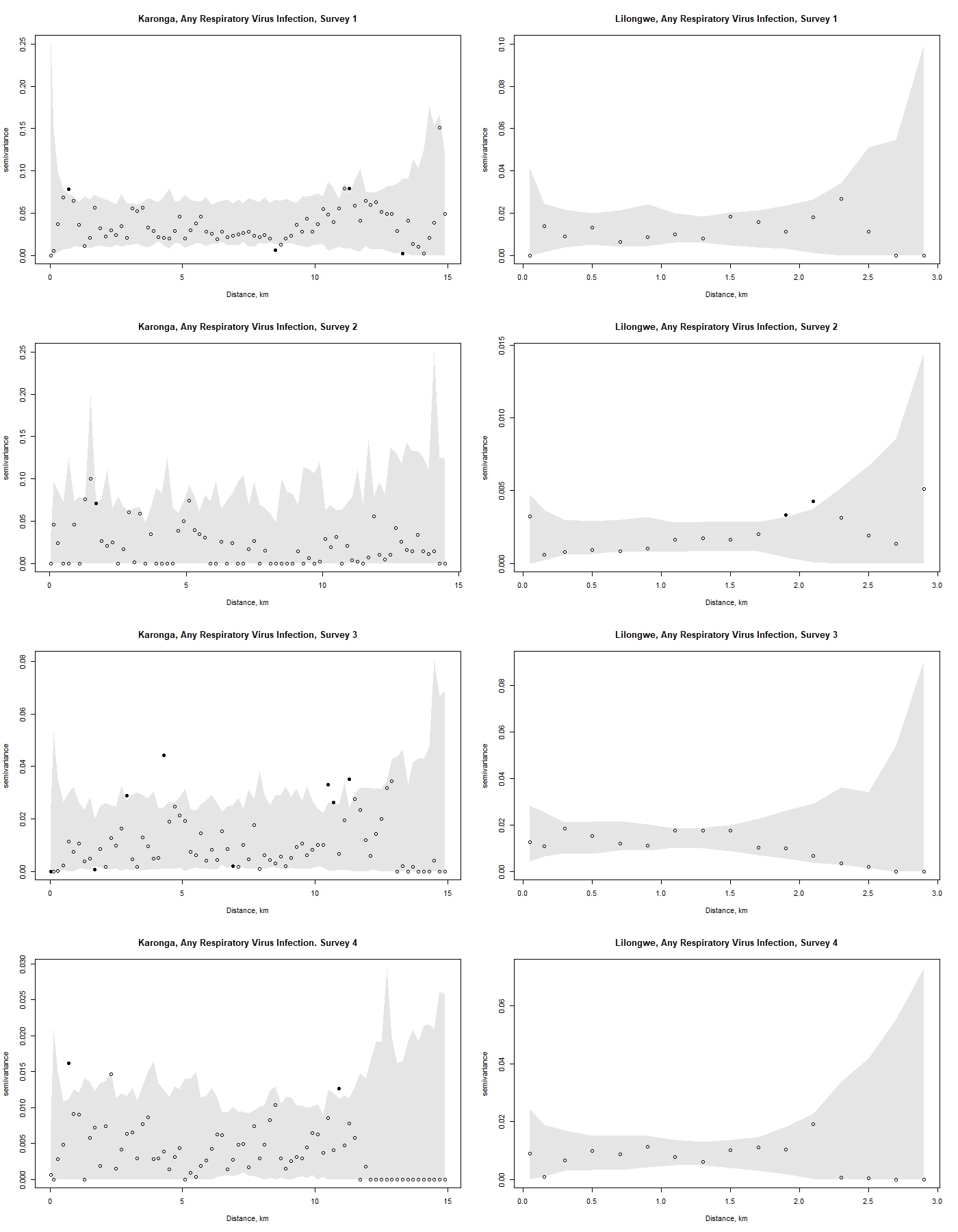


b)


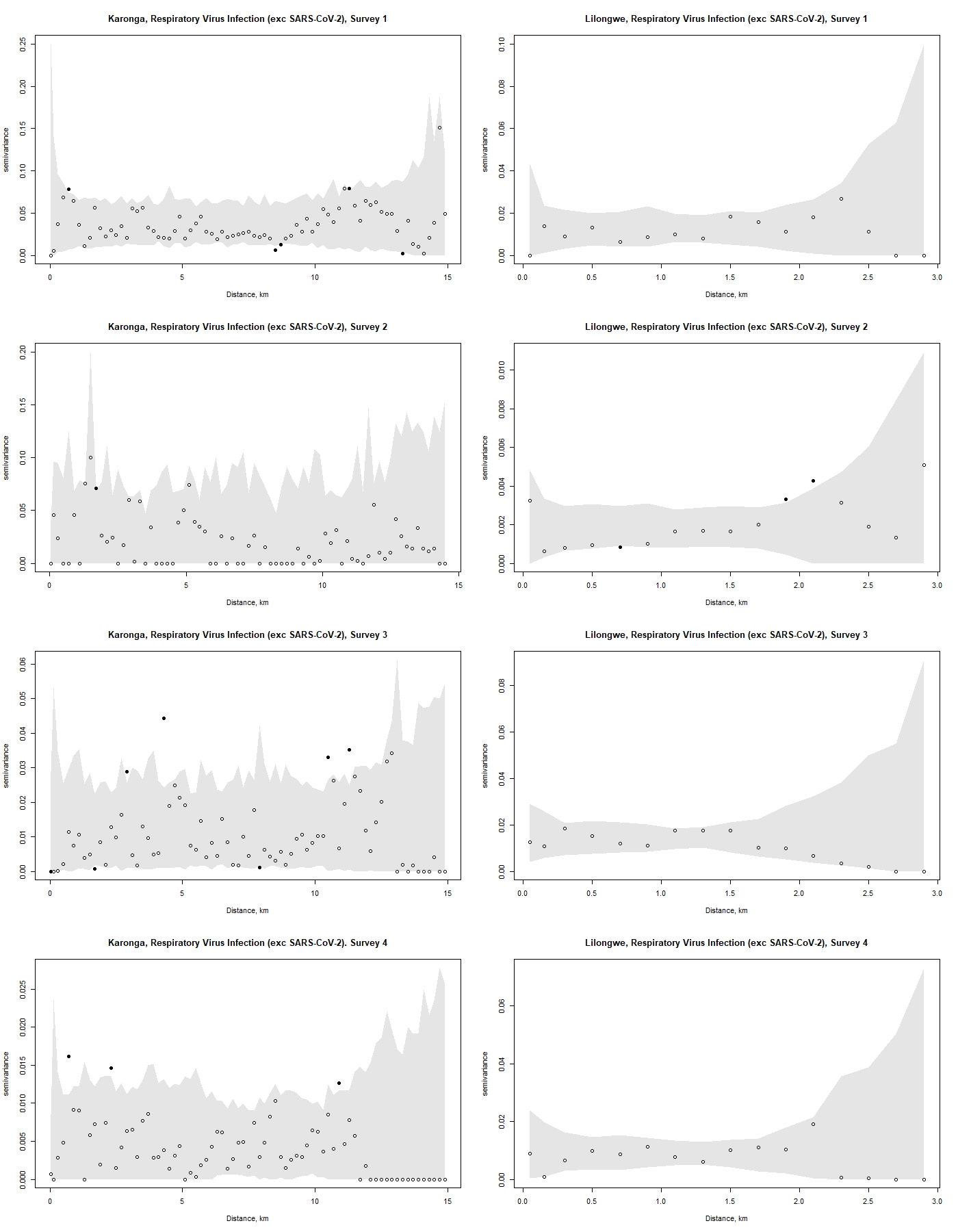


c)


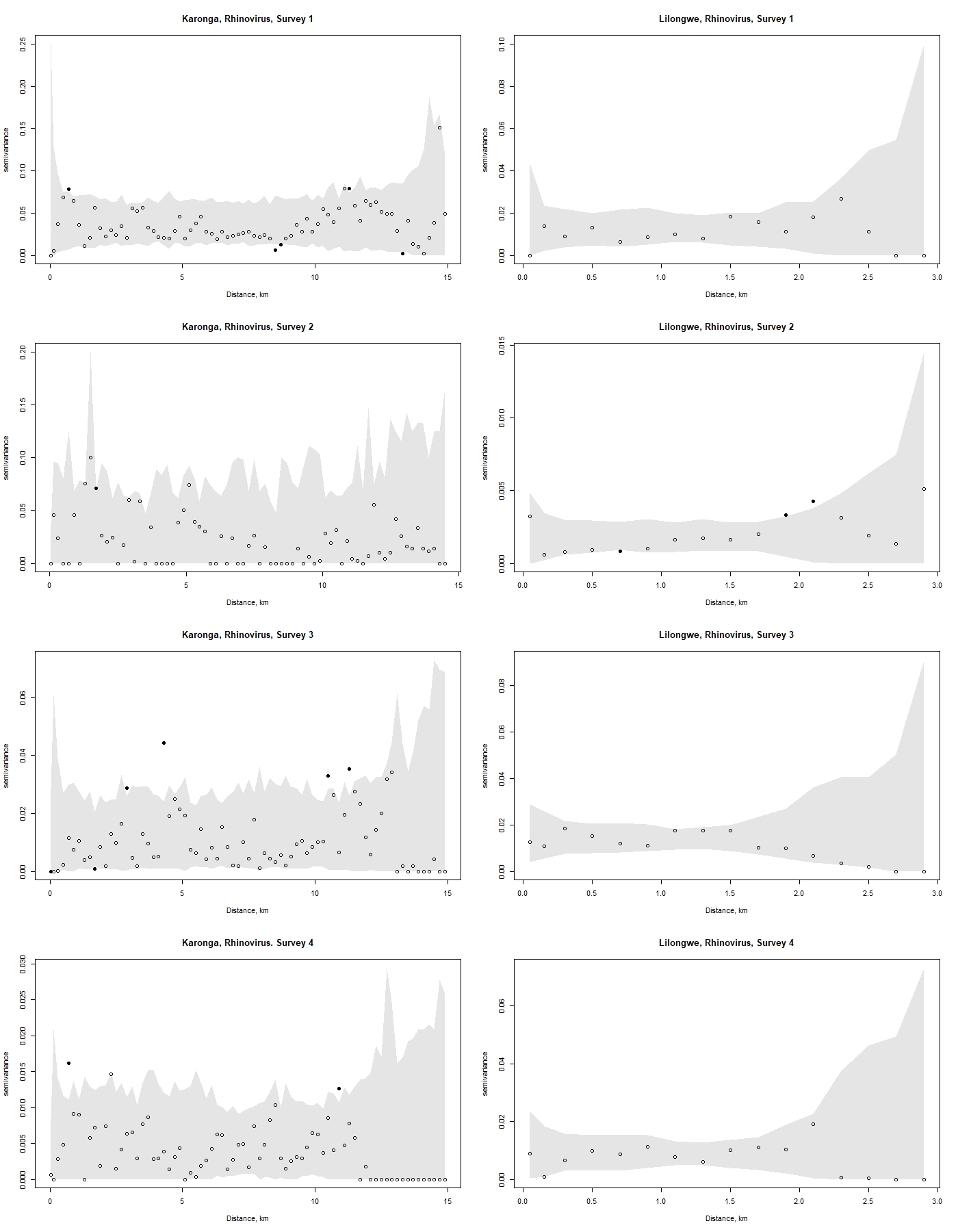


d)


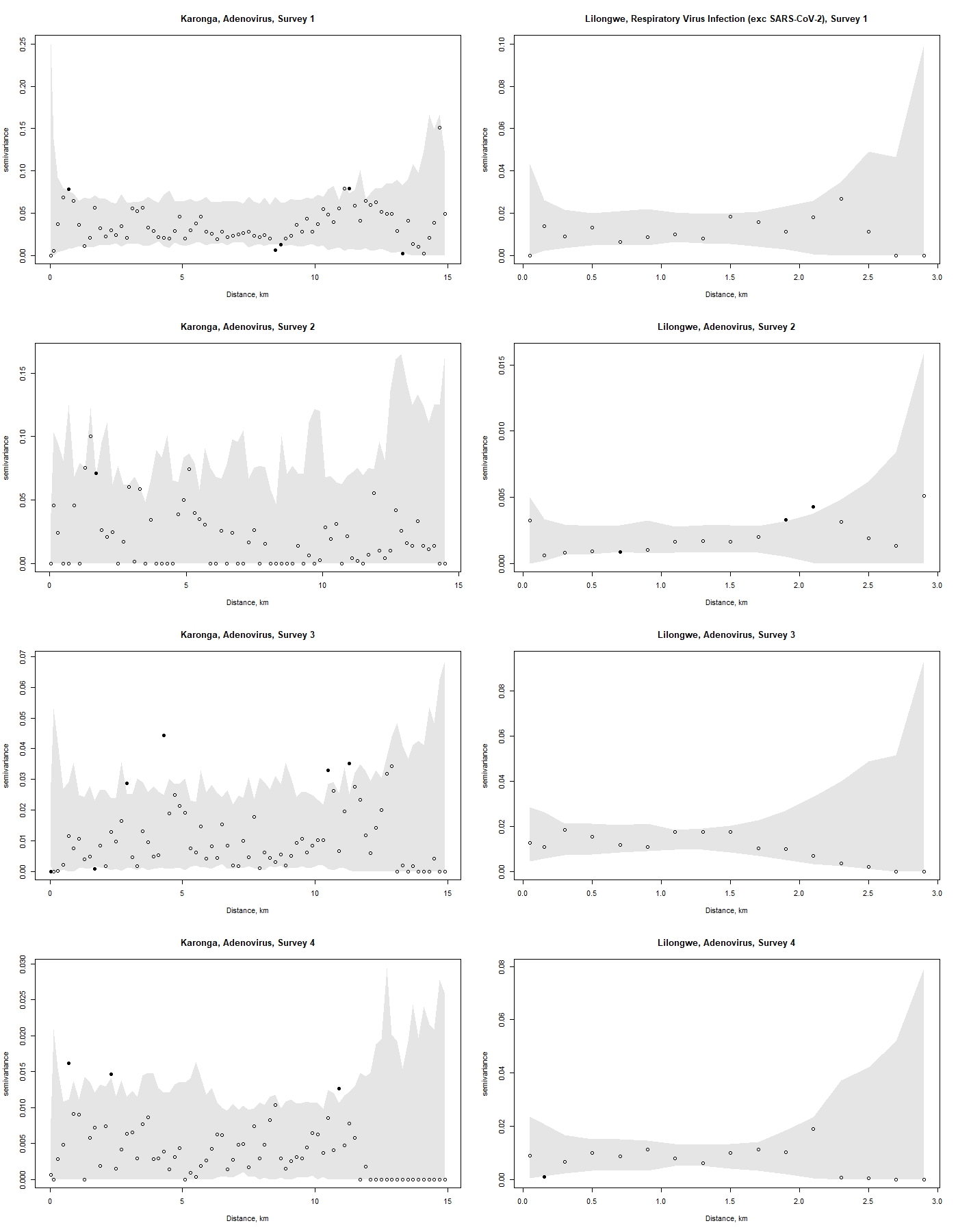

Supplement: ofad643_Supplementary_Data [file ofad643_supplementary_data.docx]
